# Co-expression of C9orf72 related dipeptide-repeats over 1000 repeat units reveals age- and combination-specific phenotypic profiles in *Drosophila*

**DOI:** 10.1186/s40478-020-01028-y

**Published:** 2020-09-07

**Authors:** Ryan J. H. West, Joanne L. Sharpe, André Voelzmann, Anna L. Munro, Ines Hahn, Richard A. Baines, Stuart Pickering-Brown

**Affiliations:** 1 Sheffield Institute for Translational Neuroscience (SITraN), University of Sheffield, 385 Glossop Road, Sheffield, S10 2HQ UK; 2 Neuroscience Institute, University of Sheffield, Sheffield, S10 2TN UK; 3 Division of Neuroscience and Experimental Psychology, Faculty of Biology, Medicine and Health, The University of Manchester, Manchester, UK; 4 Division of Molecular and Cellular Function, Faculty of Biology, Medicine and Health, The University of Manchester, Manchester, UK

**Keywords:** *Drosophila*, Frontotemporal dementia, FTD, MND, Dipeptide-repeats, C9orf72, ALS

## Abstract

A large intronic hexanucleotide repeat expansion (GGGGCC) within the C9orf72 (C9orf72-SMCR8 Complex Subunit) locus is the most prevalent genetic cause of both Frontotemporal Dementia (FTD) and Motor Neuron Disease (MND). In patients this expansion is typically hundreds to thousands of repeat units in length. Repeat associated non-AUG translation of the expansion leads to the formation of toxic, pathological Dipeptide-Repeat Proteins (DPRs). To date there remains a lack of in vivo models expressing C9orf72 related DPRs with a repeat length of more than a few hundred repeats. As such our understanding of how physiologically relevant repeat length DPRs effect the nervous system in an ageing in vivo system remains limited. In this study we generated *Drosophila* models expressing DPRs over 1000 repeat units in length, a known pathological length in humans. Using these models, we demonstrate each DPR exhibits a unique, age-dependent, phenotypic and pathological profile. Furthermore, we show co-expression of specific DPR combinations leads to distinct, age-dependent, phenotypes not observed through expression of single DPRs. We propose these models represent a unique, in vivo, tool for dissecting the molecular mechanisms implicated in disease pathology, opening up new avenues in the study of both MND and FTD.

## Introduction

Frontotemporal dementia (FTD) is a common form of early-onset dementia. It is clinically and pathologically heterogeneous and can co-occur with motor neuron disease (MND). It has a strong genetic association with up to 40% of patients presenting with a family history of disease [25]. The most prevalent genetic cause of FTD, identified to date, is an intronic hexanucleotide repeat expansion (GGGGCC) within the C9orf72 (C9orf72-SMCR8 Complex Subunit) locus [23]. In patients this expansion is typically greater than 500, and commonly thousands of, repeats in length. In unaffected individuals there are usually fewer than 25 repeats [1, 5, 31]. This mutation has also been identified as a common cause of MND, leading to the view that FTD and MND represent a clinical and pathological spectrum of a single disease [6].

The molecular mechanisms of neurodegeneration associated with the C9orf72 hexanucleotide expansion have yet to be fully elucidated. However, three potential, not necessarily mutually exclusive, hypotheses currently exist: 1/the hexanucleotide expansion leads to haploinsufficiency of the C9orf72 gene, 2/transcription of the expansion leads to the formation of toxic RNA foci and 3/non-canonical, non-AUG translation of repeat RNA leads to the formation of toxic dipeptide repeat proteins (DPRs) (Poly-GA, Poly-AP, Poly-PR, Poly-GR and Poly-GP). While it is possible that all three of these mechanisms contribute towards disease, studies have shown that C9orf72 knockout models fail to recapitulate FTD or MND phenotypes, suggesting that even though haploinsufficiency may potentiate toxic RNA and DPR gain-of-function it is unlikely to precipitate the disease in its own right [4, 10]. Although the contribution of each gain-of-function hypothesis has yet to be fully determined a number of crucial studies have demonstrated that DPRs may be the most significant driver of neurodegeneration [17, 30, 32].

Current *Drosophila* models of C9orf72 related DPRs have proven beneficial in dissecting the molecular mechanisms contributing towards neurodegeneration in FTD and MND. However, recent observations that the pathological properties of DPRs alter with length suggests that shorter repeat models may not fully recapitulate disease mechanisms [2, 19, 31]. Current *Drosophila* models may, therefore, be limited by their relatively short repeat length, the longest being 100 repeats. A number of these models also show disproportionate levels of toxicity, relative to that observed in patients, and so may not truly recapitulate disease. For example, many of these models are lethal when DPRs are expressed solely in the fly eye and require an inducible expression system to allow them to be expressed in the nervous system as viable, if short lived, adult flies. It is unclear whether this excessive toxicity is a result of the short nature of the repeats, perhaps allowing them to be more rapidly translated or to aggregate in a specific manner, or due to expression levels associated with the genomic location of the inserted transgene. The difficulties associated with the methodology of generating and maintaining long-repeats in in vivo models, coupled with the challenges of appropriate expression levels has typically precluded the use of full length DPR models in vivo. The significant toxicity of short repeat *Drosophila* models has also prevented the study of DPR toxicity during the ageing process, when continuously expressed throughout the fly’s lifetime, and when co-expressed. In this study we present novel *Drosophila* models expressing DPRs more than 1000 repeats in length. These animals exhibit age related motor decline and neurodegeneration when expressed throughout the lifetime of the fly, providing a more representative model of disease. Using these models, we demonstrate that not only do different DPRs display distinct, assay- and age- dependent, phenotypic and pathological profiles but that certain phenotypes are only observed when specific DPRs are co-expressed and flies aged.

## Materials and methods

### Drosophila stocks and maintenance

*Drosophila* were raised on standard cornmeal–yeast–sucrose medium at 25 °C on a 12 h light:dark cycle. Neuronal Synaptobrevin (nSyb)-Gal4 (RRID:BDSC_51635), Upstream activator sequence (UAS)-mCD8-GFP (RRID:BDSC_32184), Tubulin-Gal4 (RRID:BDSC_5138), OK6-Gal4 (RRID:BDSC_64199), UAS-(AP)_36_ (RRID:BDSC_58695) and UAS-(AP)_100_ (RRID:BDSC_58699) stocks were obtained from the Bloomington Drosophila Stock Center (BDSC). Glass Multimer Reporter (GMR)-Gal4 flies were a gift from Sean T. Sweeney (York, UK). nSyb-Gal4/CyO-GFP flies were a gift from Chris Elliott (York, UK). UAS-(AP)_50_ flies were a gift from Ludo Van Den Bosch (Leuven) [3]. UAS-(GR)_50_ flies were a gift from Craig Bennett (Lincoln) [7]. All 1000 repeat DPR stocks were generated as part of this study. Unless stated all experiments were performed using pan-neuronal expression (nSyb-Gal4, RRID:BDSC_51635) at 25 °C. All “wild types” are Canton S outcrossed to *w*^*1118*^. All experiments were performed using flies from at least 3 independent crosses. Experimental genotypes for each experimental figure are listed in Additional file 1: Online resource 1.

### Generation of DPR *Drosophila* lines

DPR constructs generated using semi-randomized alternative codons, described previously [2], were sub-cloned from the pEGFP-N1 vector (ClonTech) into pUASt-attB using EcoRI and XbaI restriction sites to maintain the EGFP tag. Alternative codon sequences can be found in Bennion Callister et al. (2016) [2] and Additional file 1: Online resource 2. Each dipeptide (e.g. GA) represents one repeat unit (6 base pairs, 2 amino acids). Repeat lengths for each DPR are AP:1024 repeat units, GA:1020, PR:1100, GR:1136 (See Bennion Callister et al. [2]). Each construct of ~ 1000 repeat units is followed by a C-Terminal EGFP tag, in frame. For simplicity these constructs are referred to simply as 1000 repeat DPRs throughout (e.g. UAS-(AP)_1000_-EGFP is referred to as AP1000). Previous studies from both ourselves [2] and others have shown no effect of the GFP-tag upon DPR localisation or pathology. Constructs were validated by sequencing each end of the repeat region using pUASt and EGFP primers and by agarose gel electrophoresis following restriction digest with both EcoRI and BamHI (repeat region) and EcoRI and XbaI (repeat region + EGFP). Micro-injection of the pUASt-attB-DPR-EGFP constructs into M{vas-int.DM}ZH-2A;PBac{y[+]-attP-9A}VK00005 *Drosophila* embryos allowed PhiC31-mediated integration of each UAS-DPR-EGFP into identical genomic locations. Microinjection was performed by the University of Cambridge Department of Genetics Fly Facility. Positive transformants were identified using the presence of an eye colour, resulting from by the pUASt mini-white element. Following the generation of balanced, stable stocks from each of the transformants all the lines were screened to confirm both the presence and length of the DPR construct. Southern blotting was used to identify lines carrying full length DPR constructs and to monitor DPR stability.

### Southern blotting

DNA was extracted from ~ 50 adult heads using proteinase K digestion (10 mg/ml in proteinase K buffer; 1 µl per head) and phenol–chloroform extraction. Genomic DNA was digested using Dde1 and NIaIII restriction enzymes (NEB). DNA from Canton-S flies was used as a negative control and DNA spiked with ~ 150 ng of DPR positive vector per 1 ug of genomic DNA was used as a positive control. Following agarose gel electrophoresis of the samples the gel was depurinated (10 min, 0.25 M HCl), denatured (30 min, 0.6 M NaCl, 0.2 M NaOH) and neutralised (30 min, 1.5 M NaCl, 0.5 M Tris–HCl pH8.0). Following equilibration of the gel (20 min, SSC buffer; 3 M NaCl, 300 mM Sodium citrate pH 7.4) it was assembled in the southern blotting apparatus and left to transfer onto nylon membrane overnight at room temperature. The following day the blot was disassembled and the membrane gently washed in 2 × SSC before UV fixation. The membrane was pre-hybridised in DIG easy hyb (Roche) with 3000 µg of freshly denatured salmon sperm DNA (4 h at 42 °C with rotation) before hybridisation in DIG easy hyb with 1500 µg freshly denatured salmon sperm and 75 ng of the appropriate oligo probe (GA probe: DIG-GGCAGGAGCTGGAGCTGGCGCAGGAGCTGGTGCTGGG-DIG, GR probe: DIG-AGGCAGAGGTCGTGGGAGAGGCAGGGGTCGCGGACGTGGA-DIG, AP probe: DIG-AGCACCAGCACCGGCGCCAGCTCCAGCACCAGCACCC-DIG, PR probe: DIG-AGACCCCGTCCTCGTCCTCGTCCAAGACCAAGGCCGAGGC-DIG). The membrane was hybridised overnight at 42 °C. Post hybridisation the membrane was washed (3 × 15 min 2xSSC; 0.1% Sodium dodecyl sulphate (SDS), 65 °C followed by 15 min 0.5 × SSC; 0.1% SDS), briefly rinsed in maleic acid wash buffer (DIG Wash and Block Buffer Set, Roche) and then incubated maleic acid buffer (DIG Wash and Block Buffer Set, Roche). Following blocking (DIG Wash and Block Buffer Set, (Roche) in maleic acid buffer 30 min), the membrane was incubated in anti-Digoxigenin-AP, Fab fragments (1:20,000, Sheep, 30 min, Roche, RRID:AB_2734716), washed, equilibrated in detection buffer and chemiluminescent detection performed using CPD star Chemiluminescent Substrate (Roche) on a G:box imaging unit (syngene).

### Immunoprecipitation and western blotting

Heads were isolated from ~ 1000 *Drosophila,* per genotype, pan-neuronally expressing either UAS-AP1000, UAS-GA1000, UAS-PR1000, UAS-GR1000 or UAS-mCD8-GFP under the control of nSyb-Gal4. Wild type controls were Canton-S outcrossed to *w*^*1118*^. Heads were lysed in RIPA buffer (10 mM Tris–Cl pH 8.0, 1 mM EDTA, 0.5 mM EGTA, 1% Triton X-100, 0.1% Sodium deoxycholate, 0.1%SDS, 140 mM NaCl), lysate cleared via centrifugation and filtration through 0.45 μm filters and diluted to 4 mg/ml. Lysates were incubated with pre-washed ChromoTek GFP-Trap^®^ magnetic affinity beads (30 μl, overnight 4 °C). Beads were then washed and protein eluted in 4x laemmli buffer. Samples were diluted to 1 × and run on 4–15% Mini-PROTEAN ^®^ TGX™ Precast Gels. Transfers were performed overnight (25 V, 0.02% SDS, 10% Methanol, Immobilon-P .45 μm PVDF). Primary antibodies were anti-GFP (rabbit, abcam, ab290, rabbit, abcam, ab290, preabsorbed against *Drosophila* embryos, RRID:AB_303395) and anti-GR repeat (rabbit Proteintech, 23978-1-AP**).** Secondary antibodies were HRP conjugated anti-rabbit IgG (Goat, Stratech,111-035-045-JIR). Blots were imaged using a G:box imaging unit (syngene).

### Viability and longevity assays

Experimental crosses for viability were designed to give a 50:50 ratio of offspring either expressing the DPR construct or carrying the DPR but no driver (undriven siblings) (Fig. 2a). The number of F1 offspring eclosing as adults was scored as a readout of adult viability. Driver’s used were either nSyb-Gal4 (RRID:BDSC_51635), for pan-neuronal expression, or Tubulin-Gal4 (RRID:BDSC_5138), for global expression. For longevity assays male flies were kept in vials of ~ 10 per vial and survival scored each day. Flies were transferred onto new food every 3 days. Kaplan–Meier survival curves were plotted using the survival analysis function in GraphPad Prism 8. Significance was determined using a Log-Rank (Mantel-Cox) test with Bonferroni correction for multiple comparisons.

### Ex vivo immunohistochemistry

*Drosophila* larval dissections were performed as described previously [34]. Neuromuscular Junction (NMJ) immunohistochemistry and analysis was performed as described previously [34]. Larval salivary glands were dissected in PBS and fixed for 7 min in 3.7% formaldehyde in PBS. Adult brains were dissected at 7 days post-eclosion, unless otherwise stated, and fixed for 1 h in 3.7% formaldehyde in PBS. Brains were washed 3 times in PBS-T (0.5% Triton X-100). Poly-GR and Poly-PR were labelled with anti-GFP (1:1000, rabbit, abcam, ab290, preabsorbed against *Drosophila* embryos, RRID:AB_303395). Additional primary antibodies were anti-TAR DNA-Binding Protein-43 Homologue (TBPH) (*Drosophila* TAR DNA-Binding Protein-43 (TDP-43), 1:500, Rabbit [38]), anti-elav (1:50, Mouse, DSHB, RRID:AB_2314364), anti-Cleaved Caspase 3 (1:200, rabbit, Cell Signalling Technology, 5A1E, RRID:AB_2070042), anti-bruchpilot (1:50, mouse, DSHB, RRID:AB_2314866), Cy3-conjugated anti-HRP (1:200, goat, Jackson Immuno-Research, RRID:AB_2338959). Secondary antibodies used were anti-Rabbit IgG (H + L) Alexa Fluor 488 (1:1000, RRID:AB_2576217, goat) and anti-mouse IgG (H + L) Alexa Fluor 594 (1:1000, RRID:AB_2534091, goat). Tissues were mounted in Vectashield Hardset mounting medium (RRID:AB_2336787). Imaging was performed using a Leica DM6000 B Microscope using a Hamamatsu ORCA-R^2^ C10600-10B-H camera. NMJ structural imaging was performed using the QIOPTIQ Optigrid Structured Illumination module on the same Leica DM6000 B Microscope. Cleaved Caspase 3 (CC3) quantification was performed by counting the number of CC3 positive cells throughout whole brains imaged with identical settings (2 μm z-interval), in at least 3 animals per genotype.

Quantification of the number of neurons in the central brain containing DPRs was performed by counting the number of elav positive neurons that were also GFP positive. 300 neurons within one hemisphere were counted from whole brains imaged with identical settings (2 μm z-interval), in at least 5 animals per genotype Quantification of active zones at the *Drosophila* larval NMJ was performed by counting the number of nc82/bruchpilot positive spots present within boutons of the muscle 6/7 hemi-segment A3 NMJ of third instar wandering larvae. Quantification was performed from NMJs imaged using identical settings with a 1 μm z-interval, from at least 5 animals per genotype. Salivary glands for TBPH/TDP-43 analysis were imaged using a Leica SP5 confocal microscope with an HCX PL APO CS 40.0x1.30 oil objective. Nuclear and cytoplasmic TBPH/TDP-43 fluorescence intensity was quantified relative to controls using imageJ from 25 μm z-stacks (0.5 μm z-interval) imaged using identical settings. 20 cells per animal, 3 animals per genotype from independent crosses were quantified.

### Primary neuronal cultures

Primary neuron cultures were generated following procedures described previously [21, 22, 27]. Briefly, embryos were dechorionated using bleach, selected at approximately stage 11, sterilized with ethanol and mechanically dissociated. Cells were then chemically dispersed, washed in Schneider’s medium with 20% fetal calf serum and plated onto concanavalin A (5 μg/ml) coated glass coverslips. Coverslips were kept on custom incubation chambers, where cells were grown as hanging-drop cultures at 26 °C for 3–10 days in vitro (DIV). Primary neurons were fixed in 4% paraformaldehyde (PFA) in 0.1 M phosphate-buffered saline (PBS; pH 6.8 or 7.2) for 30 min at room temperature and then washed three times in PBS with 0.3% Triton X-100 (PBT), followed by staining. Antibody staining and washes were performed in PBT using anti-tubulin (clone DM1A, mouse, Sigma-Aldrich, 1:1000, RRID:AB_477583) and anti–GFP (ab290, rabbit, abcam, 1:1000, RRID:AB_303395). Secondary antibodies were anti-rabbit Alexa Fluor 488 (1:1000, RRID:AB_2576217, goat) and Cy3-conjugated anti-mouse (1:200; Jackson Immuno-Research, RRID:AB_2315777, donkey). Culture slides were mounted in ProLong Gold.

### Histology

28 days post-eclosion *Drosophila* heads were removed and fixed in 3.7% formaldehyde in PBS + 0.1% tween, 4 °C with rotation. Heads were dehydrated and infiltrated using a graded series of ethanol:Infiltration solution (50:50, 25:75, 10:90, 0:100 × 3, 30 min 4 °C followed by 0:100 for 48 h 4 °C with rotation, (Infiltration solution: 2.5% catalyst in JB-4 Solution A (w/v), Sigma EM0100)). Heads were embedded (1:25 accelerator:infiltration solution) in polyethylene embedding moulds with embedding stubs and left to polymerise at 4 °C. Heads were sectioned at 4 μm intervals using tungsten blades on a Leica RM2255 microtome. Hematoxylin and eosin (H&E) staining and coverslipping was performed using a Leica ST5010 Autostainer XL. Sections were imaged using H&E autofluorescence in the 633 nm channel on a Leica DM6000 B Microscope using a Hamamatsu ORCA-R^2^ C10600-10B-H camera. Quantification was performed by measuring the diameter of all vacuoles within a defined 500 μm area. Measurements were taken across multiple sections covering the same region of the brain and from at least 3 animals per genotype.

### Electrophysiology

Electrophysiological recordings were carried out at room temperature in third instar wandering larvae. Larval dissection and electrophysiological recordings were performed in HL3 saline (70 mM NaCl, 5 mM KCl, 20 mM MgCl_2_ hexahydrate, 10 mM NaHCO_3_, 115 mM Sucrose, 5 mM HEPES, 1.5 mM CaCl_2_). Borosilicate glass electrodes (GC100F-10; Harvard Apparatus) were pulled to a resistance of 25-35 MΩ (Flaming brown micropipette puller, P-97; Sutter Instruments) and back filled with 3 mM KCL. Intracellular recordings were performed on muscle 6 of segments A3-4 using an AxoClamp-2B amplifier controlled by pClamp (version 10.3) with a Digidata 1322A analogue–digital converter (Molecular Devices, Axon Instruments). Frequency and amplitude of mEJP events was calculated using MiniAnalysis (v6.0.7, Synaptosoft), with mEJP events selected manually. Input Resistance (R_i_) and EJP amplitude calculated using Clampfit (v10.6, Axon Instruments).

### Motor assays: negative geotaxis

Male flies were placed individually, without anesthetisation, inside glass boiling tubes mounted on a white background. After acclimatisation the flies were banged down to the bottom of the tubes to elicit the startle-induced negative geotaxis escape behaviour. Videos were recorded until all flies reached the top or for a maximum of 90 s. Videos were processed using imageJ and custom macros. Briefly videos were batch thresholded and a custom macro used to track the movement of individual flies between frames (30 frames per second), via the MTrack2 plugin, and plot the position of the flies. These data were then used to determine the median speed of each fly. The assay was performed at 25 °C. To prevent circadian differences the assay was always performed at the same time of day, within an hour window. For co-expression of DPRs the slightly weaker pan-neuronal nSyb-Gal4 driver on the second chromosome was used. All other aspects of the assay were the same. Motor assays were performed using flies from at least 3 independent crosses per genotype.

### Genetic interaction studies: eye screens

The glass multimer reporter (GMR) Gal4 line was used to express the UAS-DPR-EGFP constructs specifically within the fly eye. Eye phenotypes were scored between 1 and 3 days post-eclosion using an 8 point classification system modified from that previously described by Pandey et al.,(2007), Ritson et al., (2010) and He et al., (2014) [8, 20, 24]. 1 point was awarded for each of the following categories: Alterations to eye size, gross morphological disruption to the eye, super-/supra-numerary interommatidial bristles, abnormal interommatidial bristle orientation, disorganisation of the ommatidial array, ommatidial fusion, pigmentation defects and the presence of melanised patches. Pharate lethality scored 9. Each fly was awarded a final score between 0 and 9. Graphs show the percentage of flies scoring each value. The mean score per genotype is also shown. Flies were scored from a minimum of 3 independent crosses per genotype. Eyes were imaged using a Zeiss Z.1 lightsheet confocal using the autofluorescence from the flies cuticle in the 488 nm wavelength. Whole flies were fixed, mounted in 1% low melting point agar and the lightsheet sample chamber filled with PBS. Samples were imaged using a 5x objective with 2x zoom.

### qRT-PCR

RNA was extracted from 10 larval brains (L3 stage) or adult heads per biological repeat. All reactions were run in duplicates for three to five independent biological repeats per genetic condition. Tissues were snap frozen and processed via standard TRIzol-chloroform extraction. cDNA and minus-reverse transcriptase controls were synthesised from 1 µg of RNA using the Quantitect cDNA synthesis kit (Qiagen), following the manufacturer’s instructions. PCR reactions for realtime qRT-PCR contained Power SYBR Green master mix (Applied Biosystems), 10 ng cDNA template (water for negative controls) and 250 nM primer mix in 20 uL volume. They were run in a Biorad CFX96 PCR machine and analysed using CFX Manager software (Biorad). To detect the expression of UAS constructs, a primer binding in the common 5’ region of all pUAST-derived transcripts was used. The expression of mEFTu1 and Rpl32 was used as reference. Relative expression levels were derived from CFX-Manager and normalised to the median of biological repeats of AP1000 (when comparing different DPR-1000s and GR-50) or AP36 (when comparing APs of different lengths). Graphs depict ΔCT values and normalised relative expression levels of biological repeats showing median values of biological repeats with 95% confidence interval range (whiskers) and data-points depict mean values from technical duplicates from each biological repeat. Data were analysed for expression differences via ANOVA with Tukey’s multiple comparison post hoc analysis on ΔCT values [37] (GraphPad Prism). Primers used were mEFTu1 forward: CATGTCCTTCATCCAACTGCA, reverse: AATGAGCTTGGTGTCTTCGCC, Rpl32 forward: GCTAAGCTGTCGCACAAATG, reverse: GTTCGATCCGTAACCGATGT and UAS forward: ACCAGCAACCAAGTAAATCAAC, reverse: ATTCCCAATTCCCTATTCAGAG.

### Statistics and graphics

Statistical analysis and generation of graphs was performed using Prism 8.3.0 (Graphpad). With the exception of graphs for qRT-PCR data, which show 95% confidence intervals, all error bars represent SEM. Figures were assembled using Adobe Illustrator (2019, version 23.1.1). The *Drosophila* “genotype builder” [26] was used with Adobe Photoshop (2019, version 21.0.1) to generate *Drosophila* “cartoons” in Fig. 2.

## Results

### Expression of stable, physiologically relevant repeat length DPRs in *Drosophila*

**Fig. 1:**
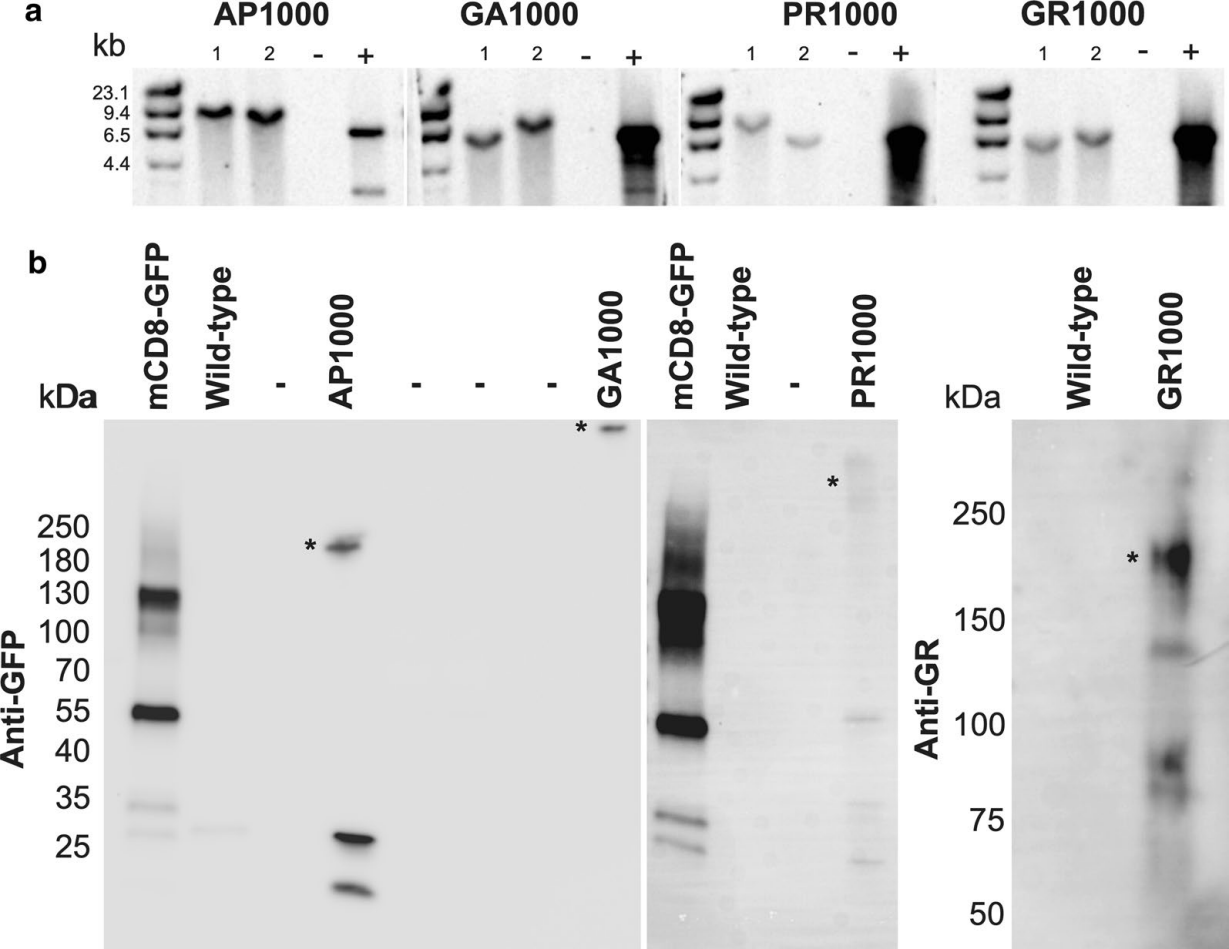
1000 repeat DPRs are stable in the *Drosophila Genome* at 12 months post-injection. **a** Southern blots showing DPR-EGFP constructs at the expected length relative to both positive controls (+) and predicted size. Lanes show ladder, two independent lines per genotype (1 and 2), negative controls (−, DNA from wild type flies) and positive controls (+, DNA from wild type flies spiked with 1000 repeat DNA). **b** Immunoblots showing DPR constructs are expressed within the *Drosophila* nervous system. DPRs were detected with either anti-GFP (AP1000, GA1000 and PR1000) or anti-GR. Asterisks show DPR bands

In order to investigate the effect physiologically relevant repeat length DPRs have in an in vivo context, *Drosophila* lines expressing DPRs with over 1000 repeats were generated (see materials and methods). The UAS/Gal4 system was used to allow cell- or tissue-specific and temporal control of DPR expression. Having observed repeat instability in a number of previous DPR models Southern blots were performed routinely, every couple of months, in order to confirm both the presence and stability of each DPR. At 12 months after the initial transformants were identified (~ 30 generations) DPR constructs were shown to be present at full length, relative to both the predicted size and the positive controls (Fig. 1a). Blots show stocks derived from two independent transformants per DPR genotype (Fig. 1a). Initial screens looking to identify which potential transformants contained full length DPRs revealed multiple lines of each genotype containing DPRs at a length comparable to the positive control (Additional file 1: Online resource 3). qRT-PCR confirmed transgene expression and revealed no significant difference in expression levels between the 4 DPR lines (AP, GA, PR, GR) (Additional file 1: Online resource 4). Immunoblotting confirmed pan-neuronal (nSyb-Gal4) expression of DPRs resulted in functional expression of each DPR transgene, resulting in proteins of comparable length to those observed previously when expressed in mammalian cell models (Fig. 1b) [2].

### Altered longevity, but not viability, in flies pan-neuronally expressing DPRs

**Fig. 2:**
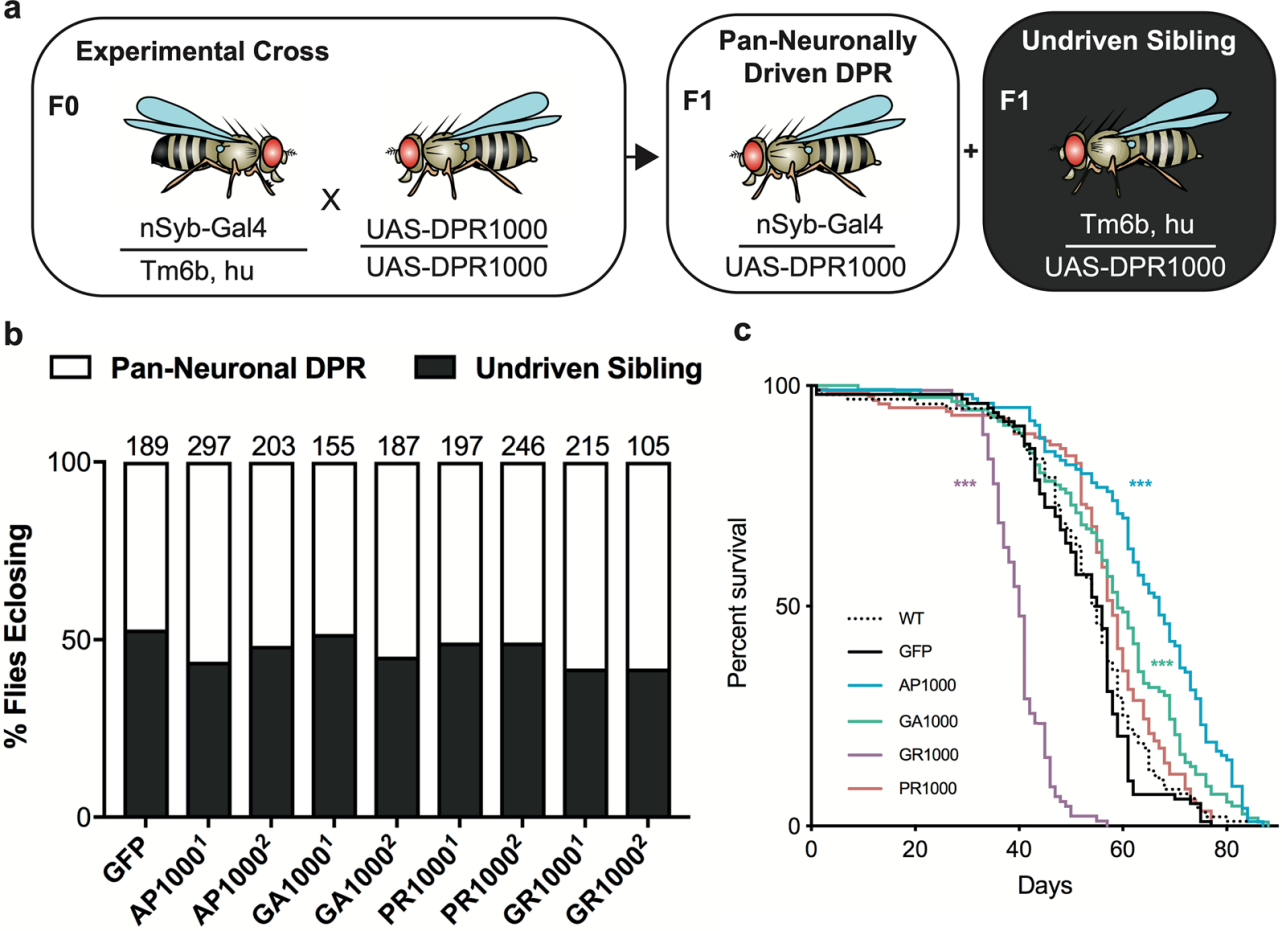
Pan-neuronal expression of 1000 repeat DPRs alters longevity but not viability. **a** Mating scheme for *Drosophila* viability assays. Flies homozygous for the UAS-GFP-DPR constructs were crossed to flies carrying the pan-neuronal driver (nSyb-Gal4) over the Tm6b balancer chromosome. Normal mendelian inheritance should result in a 50:50 ratio of flies carrying both the driver and DPR (pan-neuronally driven DPR) and those carrying only the DPR in the absence of the driver (undriven siblings). **b** pan-neuronal expression of each DPR line following the mating scheme in “**a**” shows no significant variance in viability compared to GFP (mCD8-GFP) controls or to the expected 50:50 ratio. Two independent lines of each DPR are shown. N is shown above each bar. **c** flies pan-neuronally expressing GR1000 show a significant reduction in longevity compared to wild type and control flies (Survival Log-Rank (Mantel-Cox) with Bonferroni Correction, *** p < .001 (See Additional file 1: Online resource 6)). N: wild type (WT) = 96, GFP = 98, AP = 100, GA = 111, PR = 119, GR = 90. All flies in “**b**” and “**c**” were collected from at least 3 independent crosses

Having established *Drosophila* lines containing stable, pathologically relevant repeat length DPRs we asked whether pan-neuronal expression of each DPR had a significant impact upon survival and/or longevity. Using a mating scheme designed to give a 50:50 ratio of pan-neuronally driven DPR offspring to undriven siblings (Fig. 2a) the effect of pan-neuronal DPR expression on survival to adulthood could be assessed. Pan-neuronal expression of DPRs showed no variance from typical mendelian inheritance and the 50:50 ratio of driven to un-driven siblings observed in controls (Fig. 2b). In contrast global expression of DPRs using the tubulin-Gal4 driver resulted in lethality in both AP and PR lines, as well as a reduced viability of GR flies (Additional file 1: Online resource 5). Global expression of GA showed no detrimental effect on viability. Having demonstrated pan-neuronal expression of DPRs to be adult viable we investigated whether expression of 1000 repeat length DPRs had an impact on longevity. Consistent with previous findings expression of GR significantly impaired longevity compared to wild type and GFP controls (p < .001) (Fig. 2c and Additional file 1: Online resource 6). Pan-neuronal expression of either AP1000 or GA1000 resulted in a subtle, but significant increase in longevity (p < .001 and p < .01, respectively) (Fig. 2c and Additional file 1: Online resource 6).

### DPRs show distinct localisation patterns within the Drosophila nervous system

**Fig. 3:**
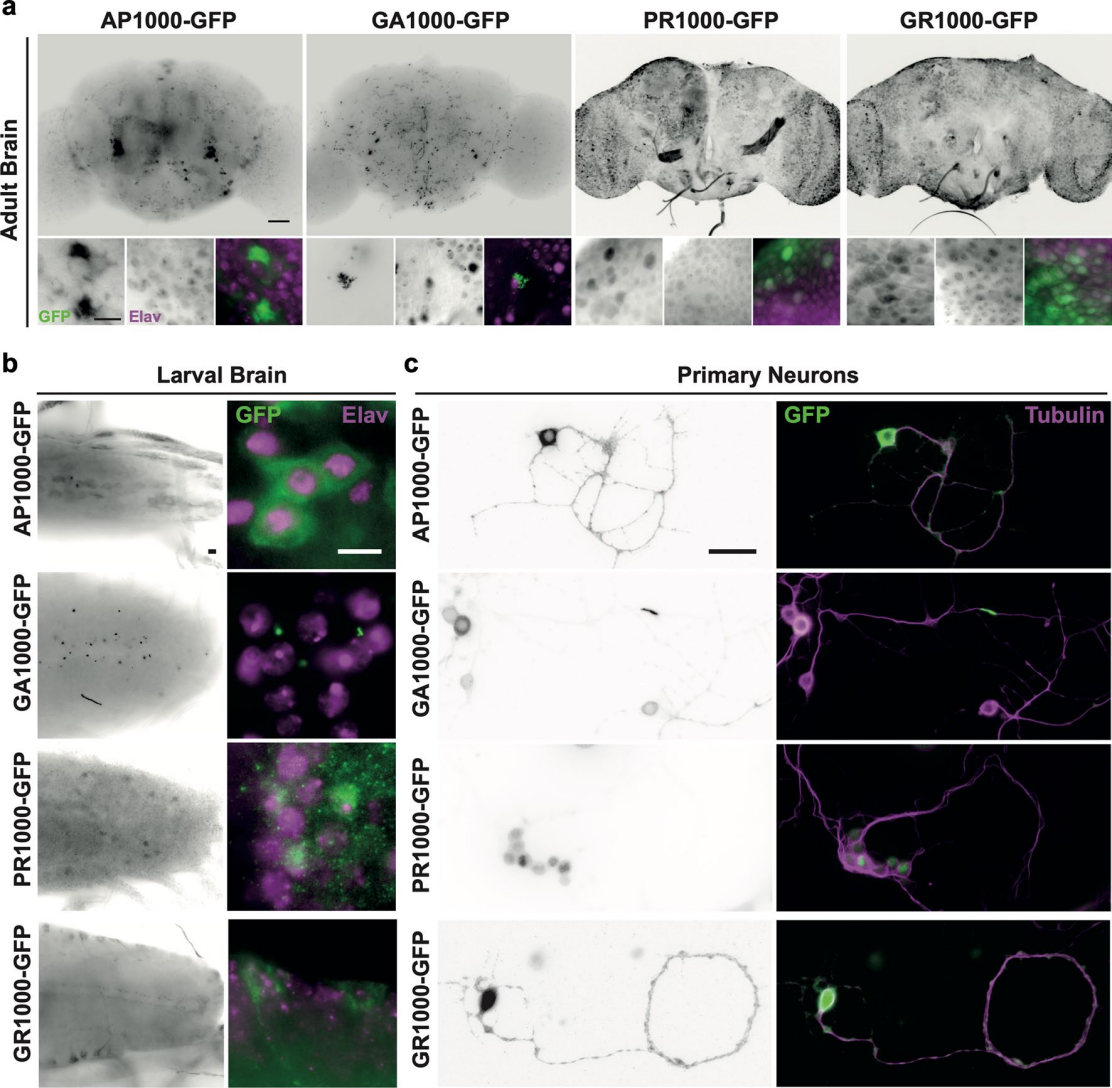
Pan-neuronal expression of DPRs reveals distinct localisation and inclusion morphology within the *Drosophila* nervous system. **a** Pan-neuronal (nSyb-Gal4) expression of EGFP-tagged DPRs in the *Drosophila* adult brain (7 days post-eclosion). Scale bars 100 μm main panel, 20 μm zoom **b** Pan-neuronal (nSyb-Gal4) expression of EGFP-tagged DPRs in the *Drosophila* larval central nervous system. Scale bars 20 μm. Neuronal nuclei are labelled with Anti-elav (magenta) in (**a**) and (**b**). **c** Primary neurons isolated from *Drosophila* embryos. Neurons were co-labelled with anti-tubulin (magenta). Scale bars 25 μm

Pan-neuronal expression of DPRs led to the localisation of DPRs throughout the CNS of both *Drosophila* adults (Fig. 3a) and larvae (Fig. 3b). While pan-neuronal expression of the GFP control (mCD8-GFP) (Additional file 1: Online resource 7) resulted in GFP localisation throughout the nervous system, each DPR showed a distinct localisation pattern. AP and GA were largely confined to the central brain, whilst PR and GR could be observed throughout both the central brain and optic lobes (Fig. 3a). DPRs were not found in every cell under the control of the pan-neuronal driver (Fig. 3 and Additional file 1: Online resource 7). While 100% of neurons (elav positive) in the adult central brain of control flies expressed mCD8-GFP DPRs were only observed in AP: 47 ± 4.07%, GA: 18.8 ± 3.28%, PR: 39.7 ± 3.16% and GR: 42.8 ± 1.4% of elav positive neurons in DPR expressing flies (n = 300 neurons per brain, N = 5 brains per genotype) (Additional file 1: Online resource 7d). DPR morphology and intracellular localisation closely resembled that seen in post-mortem patient brains [14, 18]. For example, AP and GA were predominantly observed as peri-nuclear cytoplasmic aggregates, with GA forming characteristic stellate structures. AP was also observed as diffuse granular cytoplasmic “pre-inclusion”, similar to that observed in post-mortem patient tissues [14, 18]. Both adult and larval brains revealed the occasional presence of GA inclusions within neurites. This phenotype was more obvious in cultured *Drosophila* primary neurons (Fig. 3c). Diffuse cytoplasmic staining spreading into neurites was also observed in some primary neurons expressing AP, PR and GR. PR was observed to be nuclear and cytoplasmic in both ex vivo *Drosophila* brains and primary neurons. These results are comparable to those observed previously is SH-SY5Y cells and patient tissue [2, 28]. GR showed nuclear and cytoplasmic localisation similar to that observed in post-mortem patient tissue [28]. Nucleolar DPR localisation was not typically observed.

### Altered TDP-43 localisation in DPR expressing flies

**Fig. 4:**
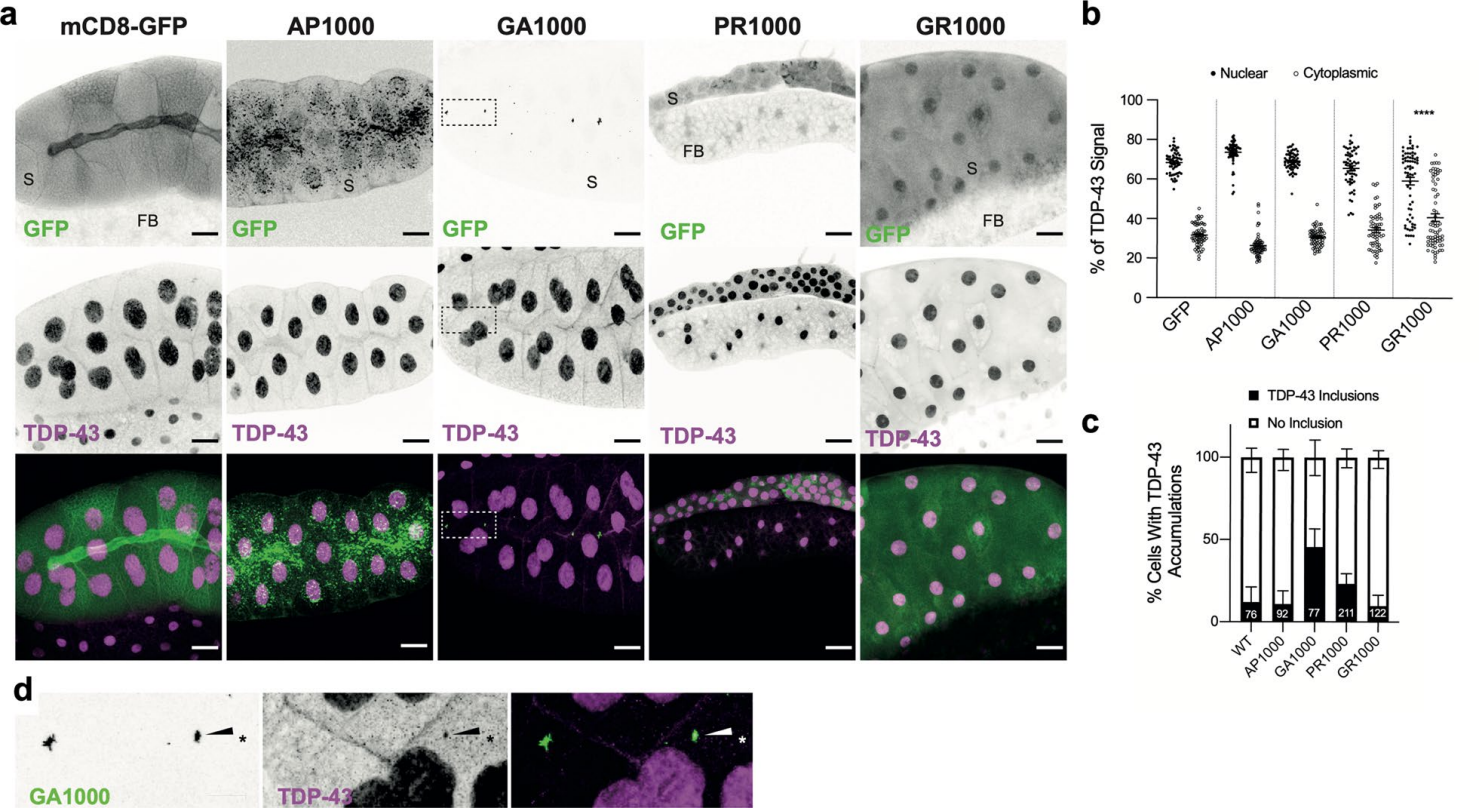
TDP-43/TBPH mislocalisation in DPR expressing *Drosophila.* **a** TDP-43/TBPH (magenta) and DPR (green) localisation in *Drosophila* Salivary glands. Scale bars 50 μm boxed region expanded in (**d**). **b** Quantification of the percentage of TDP-43/TBPH localising to the nucleus or cytoplasm (60 cells from 3 animals, from 3 independent crosses, per genotype). **c** Quantification of the percentage of DPR containing cells with insoluble TDP-43/TBPH inclusions. Total number of DPR containing cells (n) are shown on bars, total number of animals (N) = 3 per genotype. **d** co-localisation of TDP-43/TBPH and PolyGA aggregates

In addition to the accumulation of DPR aggregates, C9orf72-related FTD/MND is characterised by perturbed nuclear-cytoplasmic localisation of TDP-43. Altered localisation and aggregation of TDP-43 is a major pathological hallmark across the FTD/MND spectrum. While the pathogenic mechanisms by which TDP-43 leads to neurodegeneration remain unclear, recent studies suggest that accumulation of soluble cytosolic TDP-43 may drive toxicity in C9orf72 related FTD/MND, irrespective of the formation or insoluble TDP-43 inclusions [13]. Cytosolic accumulation of TDP-43 may be the result of, and further potentiate, nuclear cytoplasmic transport defects [11, 29, 39]. In a recent study Solomon et al. (2019) demonstrated that DPRs, not RNA accumulation, led to cytoplasmic mislocalisation of the *Drosophila* TDP-43 homologue TBPH [29]. They also demonstrated that due to their large size *Drosophila* salivary glands provide a robust model for quantification of the nuclear-cytoplasmic localisation TDP-43/TBPH [29]. This approach was used to elucidate whether 1000 repeat DPRs perturbed normal TDP-43/TBPH localisation. While expression of AP1000 and GA1000 showed no significant alteration to TDP-43/TBPH localisation, compared to GFP controls, expression of PR lead to a small, but not significant increase in the percentage of TDP-43/TBPH localising to the cytoplasm (Fig. 4a, b). Expression of GR1000 resulted in a significant mislocalisation of TDP-43/TBPH to the cytoplasm (Fig. 4a, b). Quantification of the number TDP-43/TBPH inclusions revealed only GA expression increased inclusion formation (Fig. 4c), supporting previous findings [11]. In addition, 53% of TBPH inclusions colocalised with GA aggregates (27 of 51 inclusions, 60 cells, 3 animals) (Fig. 4d). This represents 18% of GA aggregates colocalising with TDP-43/TBPH (27 of 148 aggregates). There was no clear colocalization between TDP-43/TBPH and other DPRs.

### Pan-neuronal expression of DPRs leads to neurodegeneration and cell death in the *Drosophila* central brain

**Fig. 5:**
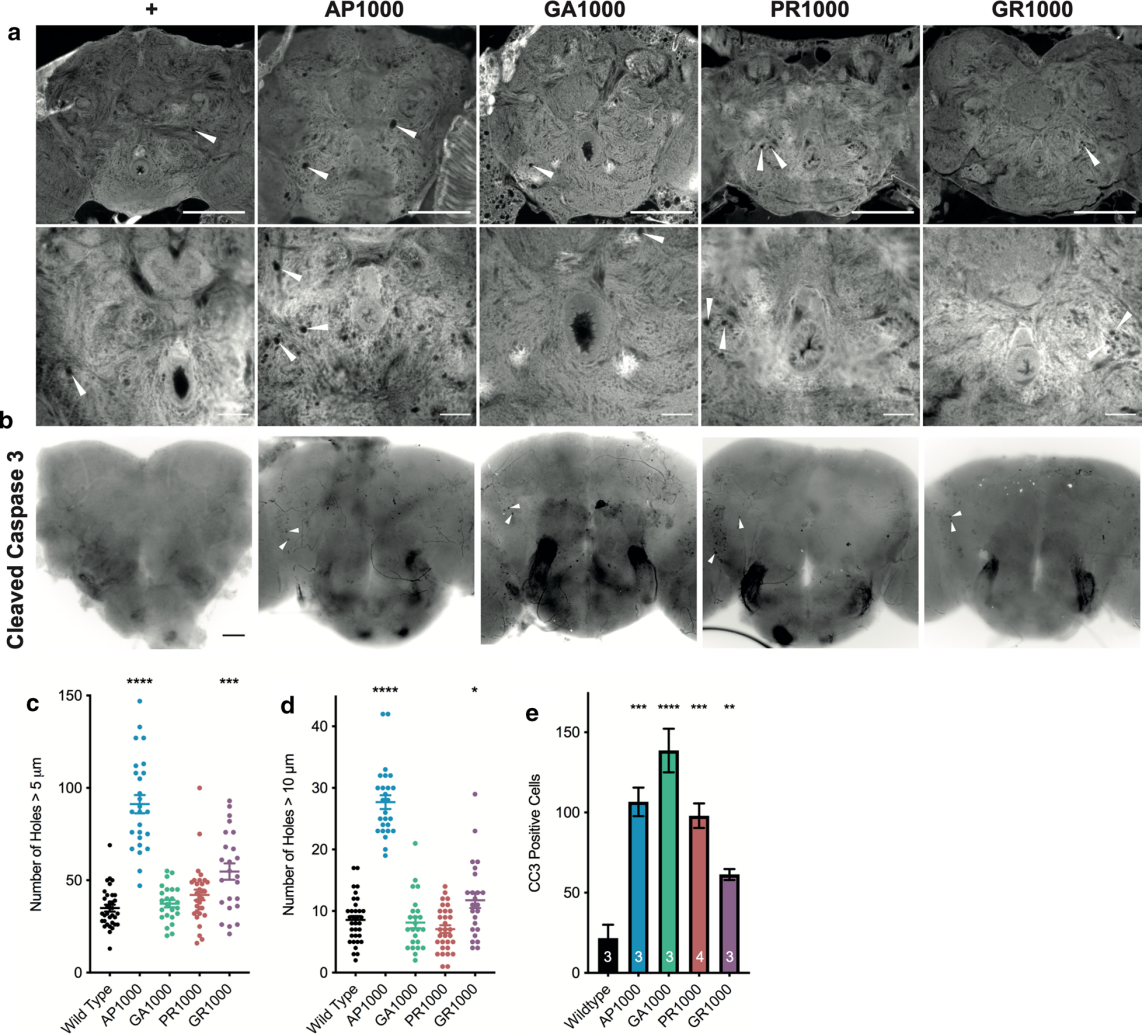
Histological analysis of *Drosophila* adult brains pan-neuronally expressing DPRs. **a** Histological sections of adult *Drosophila* brains at 28 days post-eclosion reveals vacuolar holes (examples labelled with arrows), characteristic of neurodegeneration in the *Drosophila* central nervous system. Scale bars 100 μm. **b** at 28 days post-eclosion pan-neuronal expression of DPRs results in a clear increase in the number of cells positive for the apoptotic marker cleaved caspase 3 (CC3) (arrows show examples), compared to age matched control (mCD8-GFP) flies. Scale bars 50 μm. **c**, **d** Quantification of the number of vacuoles > 5 μm (**c**) and > 10 μm (**d**) reveals a significant increase in the number of vacuoles in flies 28 days post-eclosion pan-neuronally expressing (nSyb-Gal4) UAS-AP1000 and UAS-GR1000, compared to age-matched controls (ANOVA with post hoc Dunnett’s multiple comparison to wild type controls ****p < .0001,; ***p < .001 ; *p < .05) n = 3 brains per genotype. **e** Quantification of the number of CC3 positive cells within central brain of adult *Drosophila* pan-neuronally expressing (nSyb-Gal4) DPRs at 28 days post-eclosion. (ANOVA with post hoc Dunnett’s multiple comparison to wild type controls ****p < .0001; ***p < .001; **p < .01). n is shown on bars

In order to establish whether expression of DPRs in the *Drosophila* nervous system resulted in hallmarks of neurodegeneration histological analysis was performed. Histological examination of *Drosophila* brains at 28 days post-eclosion reveals characteristic neurodegenerative vacuolar regions throughout the fly brain (Fig. 5a). Quantification reveals a significant increase in the number of vacuoles greater than 5 μm in flies expressing either AP1000 or GR1000 (p < .0001 and p < .001), compared to age matched wild type controls (Fig. 5c). AP1000 and GR1000 brains also showed a significant number of vacuoles greater than 10 μm in diameter (p < .0001 and p < .05) (Fig. 5d). The number of apoptotic cells was significantly increased in brains for all four pan-neuronally expressed DPRs, compared to age matched wild type controls (Fig. 5b, e). Taken together these results reveal all DPRs drive some degree of cell death and neurodegeneration within the *Drosophila* nervous system, although phenotypes differ between DPR species.

### DPR specific aberrations to neuronal structure and function

**Fig. 6:**
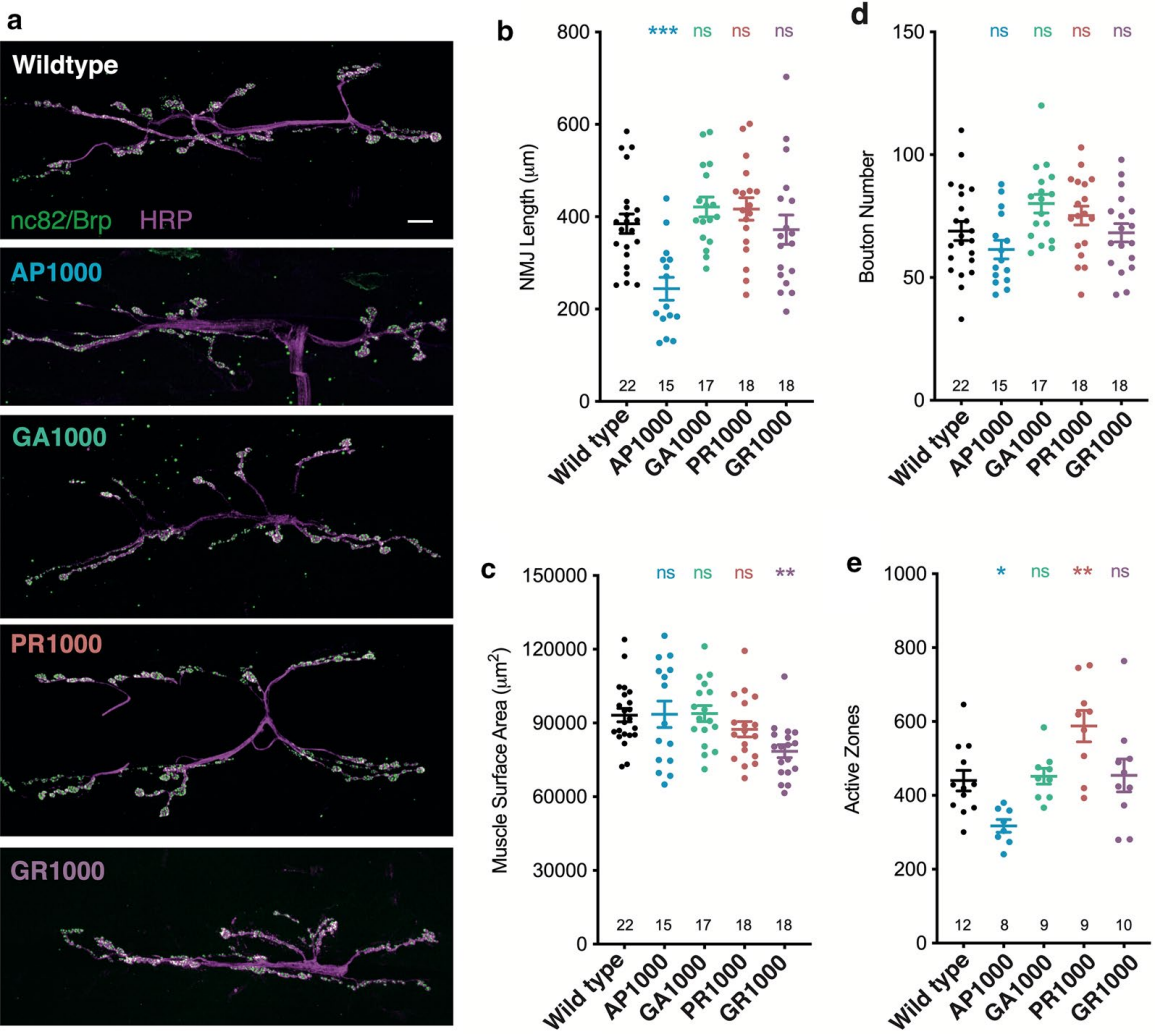
Morphological analysis of the *Drosophila* larval neuromuscular junction. **a** Micrographs showing the neuromuscular junction (NMJ) (muscle 6/7 hemi-segment A3) of third instar larvae pan-neuronally expressing (nSyb-Gal4) DPRs. Anti-HRP labels the nervous system (magenta) and anti-bruchpilot (Brp/nc82) active zones (green). Scale bars 10 μm. Quantification of **b** NMJ length, **c** muscle surface area, **d** bouton number and **e** active zone number. ANOVA with post hoc Dunnett’s multiple comparison to wild type controls ***p < .001; **p < .01; *p < .05. The number of NMJ’s analysed are shown on each graph. NMJs were quantified from at least 8 animals (N = 8) taken from at least 3 independent crosses per genotype

The *Drosophila* third instar larval neuromuscular junction (NMJ) is a well characterised model synapse with significant structural and functional similarity to vertebrate central synapses [15, 33, 34]. As a result, it has become a well-established tool for the study of neuronal structure and function in *Drosophila* models of neurodegeneration [33–36]. Morphological analysis of the NMJ at muscle 6/7 hemi-segment A3 revealed pan-neuronal expression AP1000 results in a significant reduction in NMJ length, (p < .001) coupled with a reduction in the number of bruchpilot/nc82 positive active zones (p < .05), when compared to wild type (Fig. 6). Total bouton number and muscle size was unaffected. While pan-neuronal expression of GA1000 showed no significant variance to wild type in terms of NMJ length, bouton number, muscle size or number of active zones pan-neuronal expression of GR1000 resulted in a significant reduction in muscle size (p < .01). PR1000 expression increased the number of active zones at the NMJ (p < .01) (Fig. 6). Taken together these observations suggests expression of each DPR may lead to different perturbations of molecular mechanisms, resulting in the unique phenotypic profiles observed with each DPR.

**Fig. 7:**
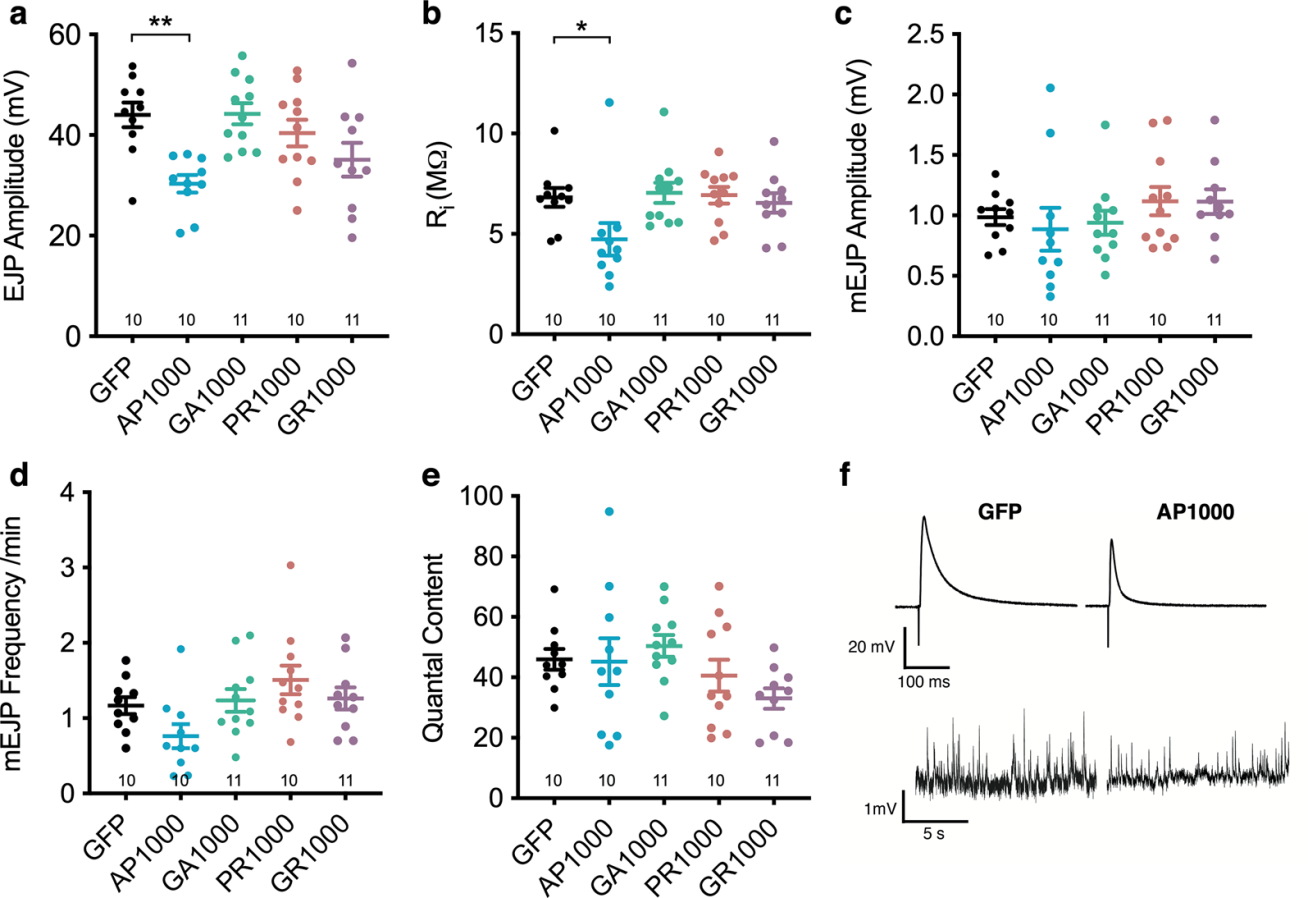
Electrophysiological analysis of DPR expressing larvae. **a** Excitatory junction potential (EJP) amplitude, **b** input resistance (Ri), **c** mini-EJP (mEJP) amplitutde, **d** mEJP frequency and **e** Quantal Content measured at muscle 6 (hemi-segment A3/4) of third instar wandering larvae pan-neuronally expressing (nSyb-Gal4) DPRs or an mCD8-GFP control. **f** representative traces showing evoked (EJP) (top traces) and spontaneous (mEJP) (bottom traces) responses in control (mCD8-GFP) and AP1000 larvae. n’s are shown on each graph. Recordings were made from at least 5 animals (N = 5) taken from at least 3 independent crosses per genotype

Previously we demonstrated a length dependent decrease in spike amplitude in differentiated SH-SY5Y cells transfected with increasing repeat lengths of PolyAP [2]. Having observed a significant decrease in both NMJ length and active zone number in *Drosophila* larvae pan-neuronally expressing AP1000 we asked whether these larvae showed impaired electrophysiological function. Pan-neuronal expression of AP1000 led to a significant (p < .01) 31% reduction in Excitatory Junction Potential (EJP) amplitude, compared to controls (Fig. 7a), consistent with the 27% reduction in active zone number observed in these animals (Fig. 6d). AP1000 expressing larvae also showed a significantly reduced input resistance (Fig. 7b) (p < .05), but no variance in quantal size (mEJP amplitude) (Fig. 7c), mini-frequency (Fig. 7d) or quantal content (Fig. 7e). Pan-neuronal expression of the other DPRs resulted in no significant variance in electrophysiological profiles when compared to controls, or each other.

### Pan-neuronal expression of arginine positive DPRs leads to an age related decline in motor function

**Fig. 8:**
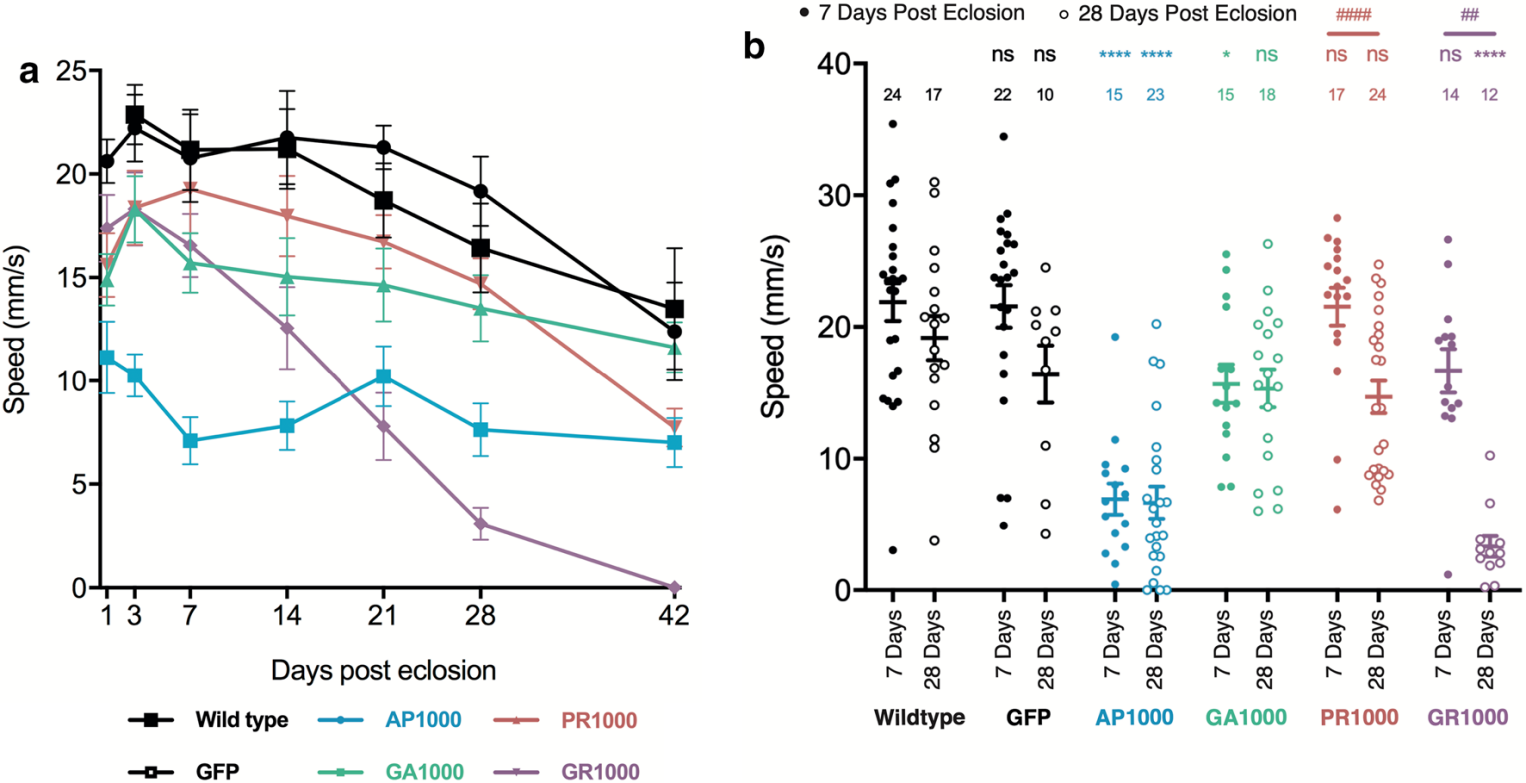
Age-related motor impairment in *Drosophila* pan-neuronally expressing DPRs. **a** Median climbing speed of adult *Drosophila* pan-neuronally expressing (nSyb-Gal4) either AP1000, GA1000, PR1000, GR1000 or mCD8-GFP controls. Outcrossed Canton S flies were used as wild type controls. Flies were assayed at 3, 7, 14, 21, 28 and 42 days post-eclosion. Error bars = SEM. At day 3 a minimum of 10 flies per genotype were assayed. The minimum number of flies at any time point was 7, resulting from lethality during the time course. For full Ns per time point see the Additional file 1: Online resource 8. **b** Median speed of adult *Drosophila* pan-neuronally expressing (nSyb-Gal4) either AP1000-GFP, GA1000-GFP, PR1000-GFP, GR1000-GFP or mCD8-GFP or outcrossed Canton S wild type controls at 7 and 28 days post-eclosion. Error bars = SEM. ANOVA with post hoc Dunnett’s multiple comparison to wild type controls ****p < .0001; *p < .05 and Tukey’s multiple comparison between groups (time points) ^####^p < .0001; ##p < .01. N’s are shown on the graph. Flies for motor assays were from at least 3 independent crosses per genotype

One of the limitations of the *Drosophila* larvae as a model system is its limited capacity in the study of ageing. In order to characterise the phenotypic profiles of each DPR in an ageing in vivo system the motor function of adult flies pan-neuronally expressing DPRs was analysed throughout the fly’s lifetime. Semi-automated tracking was used to ascertain the median speed of flies during startle-induced negative geotaxis assays, providing a proximal readout of motor function during ageing. Early in life, up to 3 days post-eclosion, only pan-neuronal expression of AP1000 showed any significant decline (p < .0001) in climbing speed, compared to age-matched controls (Fig. 8a). By 7 days post-eclosion GA1000 flies also showed a significant reduction in speed (p < .05) (Fig. 8a, b). At 28 days both AP1000 and GR1000 expressing flies showed significantly impaired climbing speed, compared to age-matched controls (Fig. 8a, b). While both AP1000 and GA1000 expressing flies showed impaired climbing by 7 days they showed no significant further reduction in motor function by 28 days (Fig. 8a, b). In contrast PR1000 and GR1000 expressing flies, which showed no variance to wild type at 7 days, displayed a significant decline in motor function from 7 to 28 days post-eclosion. As expected wild type and GFP control flies showed a slight, but non-significant, decline in climbing speed with age. The number of flies assayed at each time point in Fig. 8a is shown in Additional file 1: Online resource 8. These observations demonstrate each DPR to exhibit a distinct temporal phenotypic profile, with each capable of contributing towards impaired motor function in ageing *Drosophila*.

### Co-expression of DPRs results in novel, combination specific, phenotypic profiles

**Fig. 9:**
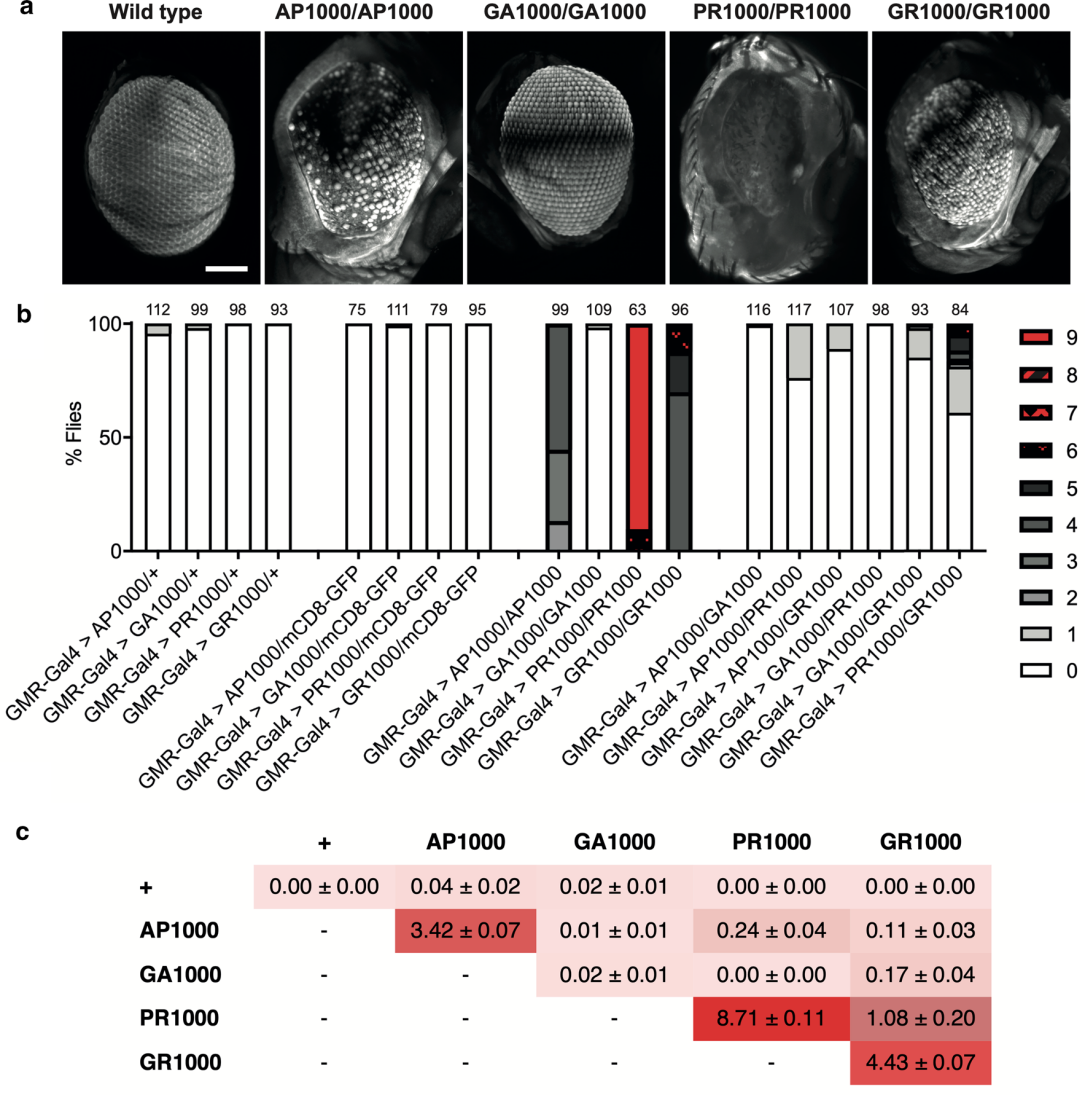
DPR-DPR modifier screens: co-expression of DPRs in the *Drosophila* Eye. **a** lighsheet micrographs showing the eye of flies homozygously expressing DPRs under the control of a single copy of the eye specific driver GMR-Gal4 (GMR-Gal4/+). Scale bars 100 μm. **b** Quantification of the eye phenotypes in flies co-expressing DPRs, compared to heterozygous and homozygous expression of single DPR species (see methods for classification scoring). Co-expression of mCD8-GFP acts as a titration control for GMR-Gal4. The number of flies scored is shown above each bar. Each genotype was scored from a minimum of 3 independent crosses. **c** Mean overall classification score (± SEM) of genotypes represented in “**b**”

In order to ascertain whether DPRs act synergistically to contribute to toxicity in these *Drosophila* models we utilised the *Drosophila* eye as a robust, high-throughput, system to screen for modification of DPR toxicity. Heterozygous expression of each DPR in the *Drosophila* eye, under the control of the eye specific driver GMR-Gal4 at 25 °C, resulted in a very mild rough eye phenotype in a small percentage of flies expressing alanine positive DPRs (Fig. 9). No flies heterozygously expressing arginine positive DPRs showed any variance to wild type (Fig. 9). Increasing the expression of the UAS-Gal4 system and therefore the dose of DPR, by raising flies at 29 °C resulted in a more severe phenotype than when expressed heterozygously at 25 °C, with all DPRs exhibiting a mild rough eye phenotype (Additional file 1: Online resource 9). Increasing the expression levels of the DPRs further through homozygous expression at 25 °C resulted in a significantly more perturbed eye phenotype in all DPRs, except for GA (Fig. 9). When homozygously expressed arginine positive DPRs showed greater toxicity than alanine positive DPRs, demonstrating the differential effect of expression levels upon the toxicity of different DPR species, at least on the eye phenotype.

While homozygous expression of most individual DPRs resulted in a significant enhancement of toxicity observed in the eye, heterozygous co-expression of two different DPRs led to a significant enhancement of the eye phenotypes only in specific combinations (Fig. 9). Toxicity associated with co-expression was typically less than seen through homozygous expression of most single DPR species. However, it was observed that co-expression of GR with any of the other DPRs resulted in a potentiation of the eye phenotype, compared to heterozygous controls or co-expression of other DPR species. Co-expression of PR and AP with each other or GR resulted in a more severe eye phenotype. Co-expression of PR and AP with GA showed no significant difference to hemizygous or homozygous expression of GA alone (Fig. 9). Co-expression of each DPR with a GFP control showed no variance to hemizygous expression alone (Fig. 9).

**Fig. 10:**
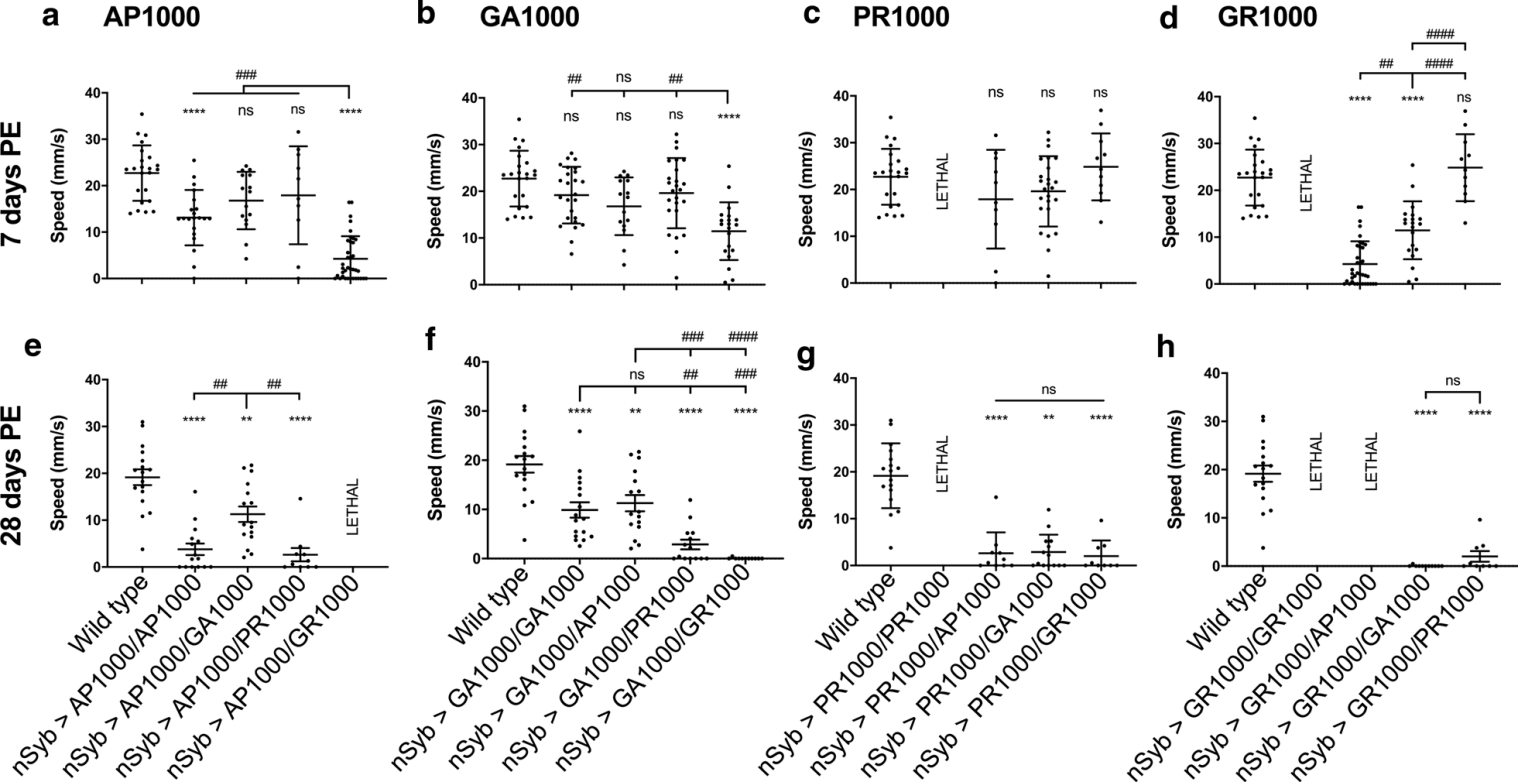
Age-related motor impairment in *Drosophila* co-expressing DPRs. **a–d** Median climbing speed of adult *Drosophila* co-expressing DPRs under the control of a single copy of the pan-neuronal driver nSyb-Gal4 (nSyb-Gal4/+) at 7 days post-eclosion. **e–h** Median speed of adult *Drosophila* co-expressing DPRs under the control of a single copy of the pan-neuronal driver nSyb-Gal4 (nSyb-Gal4/+) at 28 days post-eclosion. Error bars = SEM. ANOVA with post hoc Tukey’s multiple comparison between groups ****p < .0001; ***p < .001; **p < .01; *p < .05. Each point represents an individual fly. Motor assays were performed from at least 3 independent crosses per genotype

Having shown DPR expressing flies to display age-dependent phenotypes, and given the limitations of the fly eye as a model to look at ageing, we asked whether combining specific DPRs had any affect upon motor function and whether this alters with age (Fig. 10). At 7 days post-eclosion co-expression of either GA1000 or PR1000 with AP1000 showed no significant difference to each other, to AP1000 homozygotes, or to wild type (Fig. 10a). In contrast, flies co-expressing GR1000 with AP1000 were significantly slower than any other AP1000/DPR combination or wild types (p < .0001) (Fig. 10a). GR1000 expression also potentiated impaired climbing ability when co-expressed with GA1000, with GA1000/GR1000 flies significantly slower than wild types (p < .0001), GA1000/PR1000 (p < .01) and GA1000 homozygotes (p < .01) (Fig. 10b). GA1000/AP1000 and GA1000/PR1000 combinations showed no significant variance to wild type, at 7 days (Fig. 10b). At 7 days post-eclosion GR1000/AP1000 flies were significantly slower than those co-expressing GR1000 with either GA1000 or PR1000 (Fig. 10d). Co-expression of PR1000 with any other DPR, including GR1000, showed no significant variance from wild type (Fig. 10c). Flies pan-neuronally expressing either GR1000 or PR1000 homozygously were not adult viable, supporting our previous observations in the fly eye that homozygous expression of the arginine rich DPRs showed significant toxicity. By 28 days post-eclosion all DPR combinations showed significant impairment to motor function, compared to wild type (Fig. 10e–h). Co-expression of GR and AP was lethal by 28 days, the only non-homozygous combination to show lethality. AP1000/GA1000 and GA1000/GA1000 expressing flies were significantly faster than all other DPR combinations, suggesting these combinations are not as toxic as those combinations expressing arginine positive DPRs. Taken together these data reveal distinct age- and combination-specific phenotypic profiles in DPR expressing flies.

While performing negative geotaxis assays on aged flies co-expressing multiple DPRs it was observed that certain combinations exhibited seizure phenotypes in response to the startle “bang” stimulus. This phenotype was not observed, at any age, in flies expressing single DPRs. At 7 days post-eclosion only flies co-expressing AP1000 and GR1000 presented with seizures, with 33% penetrance. By 14 days post-eclosion AP100/GR1000 flies were observed to seizure in the absence of a stimulus. These flies failed to survive to 28 days post-eclosion. By 28 days post-eclosion seizures were also observed in GA1000/GA1000 (22%), AP1000/PR1000 (40%), GA1000/PR1000 (29%), GA1000/GR1000 (100%) and GR1000/PR1000 (71%) flies, in response to a stimulus. Seizures were never observed in AP1000/AP1000 or AP1000/GA1000 flies, at any age, suggesting seizure phenotypes are unique to specific DPR combinations.

## Discussion

In this study we establish novel *Drosophila* models of C9orf72 related dipeptide-repeats, expressing physiologically and pathologically relevant repeat lengths. These models display pathological hallmarks of C9orf72-related FTD/MND, including the formation of distinct DPR aggregates with morphological similarity to those observed in patients, as well as altered TDP-43 localisation. Using these models, we reveal each DPR to exhibit a unique, age and assay dependent, pathological and phenotypic profile. In addition, we demonstrate that specific combinations of DPRs lead to novel, age-dependent, phenotypes not observed when DPRs are expressed individually. Taken together our results provide in vivo support for our previous observations that certain DPRs may only show toxicity at a specific length, supporting the hypothesis that DPR toxicity is length dependent [2, 31]. While modelling DPRs individually has proven important in furthering our understanding of DPR toxicity our results reveal that certain phenotypes may not be observed unless DPRs are co-expressed. Whilst further investigation will be required to elucidate how DPRs interact with each other and how this leads to neuropathology we believe this model provides a useful tool for such future investigations.

The exact number or repeats required to cause pathology has yet to be fully elucidated. However, the majority of C9orf72 related FTD/MND cases reported display an expansion in the region of 500–4000 repeats [1, 5, 31]. Whether or not repeat length correlates with age of onset, disease severity and progression remain debated, as does the contribution of each DPR species to neurotoxicity. Despite a number of studies concluding alanine positive DPRs show limited toxicity, the majority of these studies look at repeat lengths below 200 repeats. Previously, we demonstrated expression of PolyAP led to a length dependent inhibition of action potential amplitude in differentiated SH-SY5Y cells [2]. Here we provide support for these findings, in a whole organism in vivo context, showing a significant decrease in evoked EJP amplitude in *Drosophila* larvae pan-neuronally expressing AP1000. Having observed both structural and functional dysfunction at the larval NMJ, motor impairment from day 1 of adulthood and significant neurodegeneration within the central brain our data suggests that, if expressed at pathologically and physiologically relevant repeat lengths throughout life, AP1000 can exhibit a significant basal level of toxicity. The same can also be said for GA1000, although in this case phenotypes manifest slightly later in life, at approximately 7 days post-eclosion. In contrast to expression of arginine positive repeats neither AP1000 or GA1000 expression displayed a significant progressive decline in motor function from the initial baseline level of impairment observed between 1 and 7 days post-eclosion. It was also observed that only GR1000 showed a significant detrimental effect upon longevity, suggesting that molecular mechanisms underpinning neurodegeneration and the characterised functional deficits do not necessarily correlate with longevity, at least at the time points characterised. As such it is clear that there are distinct phenotypic profiles associated with each DPR, with alanine positive DPRs displaying basal levels of toxicity and arginine DPRs presenting with clear age-dependent phenotypes.

A number of previous DPR fly models have been shown to present with substantial toxicity, displaying significantly impaired viability even when expressed solely in the fly eye. While these models have played an important role is dissecting molecular mechanisms contributing towards DPR toxicity to date, it may be argued that such excessive levels of toxicity are not representative of that seen in patients. Here we present a model expressing DPRs of a more physiologically relevant repeat length that can be pan-neuronal expressed throughout the entire lifetime of the fly. This is arguably more representative of disease, where patients carry the mutation throughout their lifetime with symptoms only developing with age. Through this approach we have been able to dissect subtle, age-related phenotypes including motor defects and neurodegeneration. It is important to consider that there are a number of reasons why models expressing shorter repeats may show increased toxicity. For example, it is well established that the genomic location of an inserted transgene can have a significant effect on expression levels, dependent upon chromatin state and regulator elements local to the insertion [9, 12]. As such transgenes inserted at different loci are not directly comparable. In order to elucidate whether the greater levels of toxicity observed in a number of published shorter repeat length DPR models, compared to the 1000 repeat models described here, related to higher expression levels qRT-PCR was used to directly compare expression levels of the constructs in vivo. qRT-PCR revealed expression levels in a number of previously published shorter repeat models (36, 50 and 100 repeats [3, 16]) were no greater than in the 1000 repeat models described here (Additional file 1: Online resource 4). This observation suggests the greater toxicity observed in short repeat models is not due to greater expression levels but more likely due to the intrinsic properties of the repeats. For example, one must consider that different repeat lengths may directly affect the rate of de novo synthesis, protein folding and protein–protein interactions, and that this may directly affect toxicity. This will be an important line of inquiry for further studies. Having observed dose dependent effects of toxicity both when expressing 1000 repeat DPRs in the *Drosophila* eye and upon motor function when pan-neuronally expressed, it is clear that dosage and expression levels do, however, play an important role in DPR toxicity. Our observations both in the eye and when pan-neuronally expressed also suggest that when expressed at higher doses (through homozygous expression) arginine positive DPRs are more toxic than alanine positive DPRs. However, at lower expression levels, particularly in younger flies, alanine DPRs appear to show more severe phenotypes than those expressing arginine positive DPRs. This data suggests the importance of both expression levels and age when studying DPR toxicity. While it is currently not possible to determine whether the expression levels of DPRs in our models are comparable to those observed in patients the ability to express DPRs over 1000 repeats in length pan-neuronally throughout the fly’s lifetime suggests the models described here may represent more physiologically relevant models of DPRs. Having shown that each DPR shows distinct localisation throughout the nervous system and that DPRs are not seen in every cell under the control of the pan-neuronal driver it may also be important to consider whether each DPR has an effect on different neuronal-subtypes and whether this effect is autonomous or non-autonomous.

The observation that co-expression of arginine and alanine positive DPR species with each other showed a more significant, age related, decline in motor function than when either arginine or alanine DPRs were co-expressed (e.g. GA + AP, GR + PR) suggests that the progressive degeneration seen in arginine positive DPRs may be potentiated by a basal level of dysfunction observed through expression of alanine positive species. Impaired motor function was most pronounced through co-expression of GR and AP, with GR1000/AP1000 expressing flies significantly slower than any other combination at 7 days post-eclosion. This combination was also the only one to show stimulus induced seizures at 7 days and seizures in the absence of a stimulus at any age. GR1000/AP1000 flies also showed premature lethality by 28 days, not seen in any other non-homozygous combination. Taken together this data suggest that AP and GR may show the most significant pathological interaction. Further investigation will be essential to dissect the molecular mechanisms through which this interaction may occur and determine the effect this has on the ageing nervous system. The identification that specific DPR combinations elicit distinct phenotypic profiles, including the development of age-related seizure phenotypes suggests that studying DPRs in combination may prove important in dissecting the molecular mechanisms driving neuronal dysfunction in FTD/MND.

To date there remains a lack of in vivo models expressing C9orf72 related dipeptide repeats with a repeat length of more than a few hundred repeats. Whilst these models have proven incredibly useful in dissecting the molecular mechanisms contributing towards neurodegeneration in FTD and MND they may not truly recapitulate the functional and pathological features of longer DPRs observed in patients. The model presented here not only highlights the potential importance of repeat length, but also of studying DPRs in combination and at different ages

## Supplementary information

**Additional file 1:** **Online Resource 1**. Full Genotype Lists for Each Figure. (Microsoft Word Document (.docx)).** Online Resource 2**. DPR Sequences. (Microsoft Word Document (.docx)).** Online Resource 3**. Southern Blots of all DPR lines at 3 months. (Microsoft Word Document (.docx)).** Online Resource 4**. qRT-PCR graphs showing comparative expression levels between 1000 repeat DPR lines and shorter repeat models. (Microsoft Word Document (.docx)).** Online Resource 5**. Viability Assays for global DPR expression. (Microsoft Word Document (.docx)).** Online Resource 6**. Survival Log-Rank (Mantel-Cox) with Bonferroni Correction for longevity assays. (Microsoft Word Document (.docx)).** Online Resource 7**. Localisation of GFP controls and quantification of GFP positive neurons. (Microsoft Word Document (.docx)).** Online Resource 8**. Number of flies assayed at each time point for longitudinal negative geotaxis assays. (Microsoft Word Document (.docx)).** Online Resource 9**. Quantification of the eye phenotype in flies expressing DPRs in the Drosophila eye (GMR-Gal4) at 29°C. (Microsoft Word Document (.docx)).

## Data Availability

Data generated or analysed during this study are included in this published article [and its supplementary information files]. Datasets generated and/or analysed during the current study are available from the corresponding author on reasonable request.

## Abbreviations

AP: alanine proline
DPR(s): dipeptide repeat(s)
FTD: frontotemporal dementia
GA: glycine alanine
GMR: glass multimer reporter
GR: glycine arginine
MND: motor neurone disease
nSyb: neuronal synaptobrevin
PR: proline arginine
UAS: upstream activator sequence
