# Supplementary material for: Co-expression of C9orf72 related dipeptide-repeats over 1000 repeat units reveals age- and combination-specific phenotypic profiles in Drosophila
Source: Acta Neuropathol Commun. 2020 Sep 7;8:158. doi: 10.1186/s40478-020-01028-y (PMC7487709; doi:10.1186/s40478-020-01028-y)
Supplement: Supplementary file 1 — Additional file 1: Online Resource 1. Full Genotype Lists for Each Figure. (Microsoft Word Document (.docx)). Online Resource 2. DPR Sequences. (Microsoft Word Document (.docx)). Online Resource 3. Southern Blots of all DPR lines at 3 months. (Microsoft Word Document (.docx)). Online Resource 4. qRT-PCR graphs showing comparative expression levels between 1000 repeat DPR lines and shorter repeat models. (Microsoft Word Document (.docx)). Online Resource 5. Viability Assays for global DPR expression. (Microsoft Word Document (.docx)). Online Resource 6. Survival Log-Rank (Mantel-Cox) with Bonferroni Correction for longevity assays. (Microsoft Word Document (.docx)). Online Resource 7. Localisation of GFP controls and quantification of GFP positive neurons. (Microsoft Word Document (.docx)). Online Resource 8. Number of flies assayed at each time point for longitudinal negative geotaxis assays. (Microsoft Word Document (.docx)). Online Resource 9. Quantification of the eye phenotype in flies expressing DPRs in the Drosophila eye (GMR-Gal4) at 29°C. (Microsoft Word Document (.docx)). [file 40478_2020_1028_MOESM1_ESM.pdf]

Online Resources

Co-expression of C9orf72 related Dipeptide-repeats over 1000 repeat units reveals age- and combination-specific phenotypic profiles in *Drosophila*

Acta Neuropathologica Communications

Ryan J.H. West[1,2,5] †, Joanne L. Sharpe[3,5], André Voelzmann[4], Anna L. Munro[3], Ines Hahn[4], Richard A. Baines[3] and Stuart Pickering-Brown[3]

[1] Sheffield Institute for Translational Neuroscience (SITraN), University of Sheffield, 385 Glossop Road, Sheffield, UK.

[2] Neuroscience Institute, University of Sheffield, Sheffield, S10 2TN, UK

[3] Division of Neuroscience & Experimental Psychology, Faculty of Biology, Medicine and Health, The University of Manchester, UK

[4] Division of Molecular & Cellular Function, Faculty of Biology, Medicine and Health, The University of Manchester, UK

[5] Authors contributed equally

r.j.west@sheffield.ac.uk

Online Resource 1. Full Genotype List For Each Figure

Figure 1a

;; UAS-AP1000^{1}/Tm6b (Undriven Stock)
;; UAS-AP1000^{2}/Tm6b (Undriven Stock)
;; UAS-GA1000^{1}/Tm6b (Undriven Stock)
;; UAS-GA1000^{2}/Tm6b (Undriven Stock)
;; UAS-PR1000^{1}/Tm6b (Undriven Stock)
;; UAS-PR1000^{2}/Tm6b (Undriven Stock)
;; UAS-GR1000^{1}/Tm6b (Undriven Stock)
;; UAS-GR1000^{2}/Tm6b (Undriven Stock)
Wild-type (Canton S, w^{1118} outcross)

Figure 1b

;;nsyb-gal4/UAS-AP1000^{1}
;;nsyb-gal4/UAS-GA1000^{1}
;;nsyb-gal4/UAS-PR1000^{1}
;;nsyb-gal4/UAS-GR1000^{1}
;;nsyb-gal4/UAS-mCD8-GFP
Wild-type (Canton S, w^{1118} outcross)

Figure 2a-b

;;nsyb-gal4/UAS-AP1000^{1}
;;nsyb-gal4/UAS-AP1000^{2}
;;nsyb-gal4/UAS-GA1000^{1}
;;nsyb-gal4/UAS-GA1000^{2}
;;nsyb-gal4/UAS-PR1000^{1}
;;nsyb-gal4/UAS-PR1000^{2}
;;nsyb-gal4/UAS-GR1000^{1}
;;nsyb-gal4/UAS-GR1000^{2}
;;nsyb-gal4/UAS-mCD8-GFP
;;nsyb-gal4/Tm6b (undriven siblings of each cross)

Figure 2c,3,5,6,7,8.

;;nsyb-gal4/UAS-AP1000^{1}
;;nsyb-gal4/UAS-GA1000^{1}
;;nsyb-gal4/UAS-PR1000^{1}
;;nsyb-gal4/UAS-GR1000^{1}
;;nsyb-gal4/UAS-mCD8-GFP
Wild-type (Canton S, w^{1118} outcross)

Figure 4

;OK6-gal4/+; UAS-mCD8-GFP/+
;OK6-gal4/+;UAS-AP1000^{1}/+

;OK6-gal4/+;UAS-GA1000^{1}/+
;OK6-gal4/+;UAS-PR1000^{1}/+
;OK6-gal4/+;UAS-GR1000^{1}/+

Figure 9.

;GMR-gal4/+;UAS-AP1000^{1}/+
;GMR-gal4/+;UAS-GA1000^{1}/+
;GMR-gal4/+;UAS-PR1000^{1}/+
;GMR-gal4/+;UAS-GR1000^{1}/+
;GMR-gal4/+;UAS-AP1000^{1}/ mCD8-GFP
;GMR-gal4/+;UAS-GA1000^{1}/ mCD8-GFP
;GMR-gal4/+;UAS-PR1000^{1}/ mCD8-GFP
;GMR-gal4/+;UAS-GR1000^{1}/ mCD8-GFP
;GMR-gal4/+;UAS-AP1000^{1}/ UAS-AP1000^{1}
;GMR-gal4/+;UAS-GA1000^{1}/ UAS-GA1000^{1}
;GMR-gal4/+;UAS-PR1000^{1}/ UAS-PR1000^{1}
;GMR-gal4/+;UAS-GR1000^{1}/ UAS-GR1000^{1}
;GMR-gal4/+;UAS-AP1000^{1}/ UAS-GA1000^{1}
;GMR-gal4/+;UAS-AP1000^{1}/ UAS-PR1000^{1}
;GMR-gal4/+;UAS-AP1000^{1}/ UAS-GR1000^{1}
;GMR-gal4/+;UAS-GA1000^{1}/ UAS-PR1000^{1}
;GMR-gal4/+;UAS-GA1000^{1}/ UAS-GR1000^{1}
;GMR-gal4/+;UAS-PR1000^{1}/ UAS-GR1000^{1}

Figure 10.

Wild-type (Canton S, w^{1118} outcross)
; nsyb-gal4/+; UAS-AP1000^{1}/ UAS-AP1000^{1}
; nsyb-gal4/+; UAS-AP1000^{1}/ UAS-GA1000^{1}
; nsyb-gal4/+; UAS-AP1000^{1}/ UAS-PR1000^{1}
; nsyb-gal4/+; UAS-AP1000^{1}/ UAS-GR1000^{1}
; nsyb-gal4/+; UAS-GA1000^{1}/ UAS-GA1000^{1}
; nsyb-gal4/+; UAS-GA1000^{1}/ UAS-AP1000^{1}
; nsyb-gal4/+; UAS-GA1000^{1}/ UAS-PR1000^{1}
; nsyb-gal4/+; UAS-GA1000^{1}/ UAS-GR1000^{1}
; nsyb-gal4/+; UAS-PR1000^{1}/ UAS-PR1000^{1}
; nsyb-gal4/+; UAS-PR1000^{1}/ UAS-AP1000^{1}
; nsyb-gal4/+; UAS-PR1000^{1}/ UAS-GA1000^{1}
; nsyb-gal4/+; UAS-PR1000^{1}/ UAS-GR1000^{1}
; nsyb-gal4/+; UAS-GR1000^{1}/ UAS-GR1000^{1}
; nsyb-gal4/+; UAS-GR1000^{1}/ UAS-AP1000^{1}
; nsyb-gal4/+; UAS-GR1000^{1}/ UAS-GA1000^{1}
; nsyb-gal4/+; UAS-GR1000^{1}/ UAS-PR1000^{1}

AP

gaattcggatgtagaccATG[GCCCCTGCTCCTGCCCCTGCGCCGGCTCCAGCTCCAGCGCCTGCACCAG
CCCCTGCTCCTGCACCAGCACCAGCACCGGCGCCAGCTCCAGCACCAGCACCCGCTCCTGCTCCTGCTCC
CGCTCCAGCACCAGCGCCTGCTCCTGCTCCGGCCCCAGCTCCTGCTCCAGCGCCCGCGCCGGCCCCGGCC
CCAGCACCGGCCCCAGCTCCGgcccCTGCTCCTGCCCCTGCGCCGGCTCCAGCTCCAGCGCCTGCACCAG
CCCCTGCTCCTGCACCAGCACCAGCACCGGCGCCAGCTCCAGCACCAGCACCCGCTCCTGCTCCTGCTCC
CGCTCCAGCACCAGCGCCTGCTCCTGCTCCGGCCCCAGCTCCTGCTCCAGCGCCCGCGCCGGCCCCGGCC
CCAGCACCGGCCCCAGCTCCGGCCCCT]gtcttcCAAcgggatccaccggtcgccaccatggtgagcaag
ggcgaggagctgttcaccggggtggtgcccatcctggtcgagctggacggcgacgtaaacggccacaagt
tcagcgtgtccggcgagggcgagggcgatgccacctacggcaagctgaccctgaagttcatctgcaccac
cggcaagctgcccgtgccctggcccaccctcgtgaccaccctgacctacggcgtgcagtgcttcagccgc
taccccgaccacatgaagcagcacgacttcttcaagtccgccatgcccgaaggctacgtccaggagcgca
ccatcttcttcaaggacgacggcaactacaagacccgcgccgaggtgaagttcgagggcgacaccctggt
gaaccgcatcgagctgaagggcatcgacttcaaggaggacggcaacatcctggggcacaagctggagtac
aactacaacagccacaacgtctatatcatggccgacaagcagaagaacggcatcaaggtgaacttcaaga
tccgccacaacatcgaggacggcagcgtgcagctcgccgaccactaccagcagaacacccccatcggcga
cggccccgtgctgctgcccgacaaccactacctgagcacccagtccgccctgagcaaagaccccaacgag
aagcgcgatcacatggtcctgctggagttcgtgaccgccgccgggatcactctcggcatggacgagctgt
acaagtaaagcggccgcgactctaga

GA

gaattcggatgtagaccATG[GGTGCTGGCGCGGGAGCAGGCGCTGGTGCTGGTGCAGGAGCGGGTGCGG
GAGCTGGTGCCGGCGCAGGAGCTGGAGCTGGCGCAGGAGCTGGTGCTGGGGCTGGTGCCGGTGCCGGTGC
TGGAGCTGGAGCAGGAGCAGGCGCGGGTGCAGGGGCCGGAGCGGGTGCTGGTGCTGGTGCTGGAGCGGGA
GCGGGCGCTGGAGCCGGCGCCGGTGCTGGCGCGGGAGCAGGCGCTGGTGCTGGTGCAGGAGCGGGTGCGG
GAGCTGGTGCCGGCGCAGGAGCTGGAGCTGGCGCAGGAGCTGGTGCTGGGGCTGGTGCCGGTGCCGGTGC
TGGAGCTGGAGCAGGAGCAGGCGCGGGTGCAGGGGCCGGAGCGGGTGCTGGTGCTGGTGCTGGAGCGGGA
GCGGGCGCTGGAGCCGGCGCCGGTGCT]gtcttccaacgggatccaccggtcgccaccatggtgagcaag
ggcgaggagctgttcaccggggtggtgcccatcctggtcgagctggacggcgacgtaaacggccacaagt
tcagcgtgtccggcgagggcgagggcgatgccacctacggcaagctgaccctgaagttcatctgcaccac
cggcaagctgcccgtgccctggcccaccctcgtgaccaccctgacctacggcgtgcagtgcttcagccgc
taccccgaccacatgaagcagcacgacttcttcaagtccgccatgcccgaaggctacgtccaggagcgca
ccatcttcttcaaggacgacggcaactacaagacccgcgccgaggtgaagttcgagggcgacaccctggt
gaaccgcatcgagctgaagggcatcgacttcaaggaggacggcaacatcctggggcacaagctggagtac
aactacaacagccacaacgtctatatcatggccgacaagcagaagaacggcatcaaggtgaacttcaaga
tccgccacaacatcgaggacggcagcgtgcagctcgccgaccactaccagcagaacacccccatcggcga
cggccccgtgctgctgcccgacaaccactacctgagcacccagtccgccctgagcaaagaccccaacgag
aagcgcgatcacatggtcctgctggagttcgtgaccgccgccgggatcactctcggcatggacgagctgt
acaagtaaagcggccgcgactctaga

PR

gaattcggatgtagaccATG[CCGCGACCTCGACCGCGGCCACGCCCACGCCCTCGGCCCAGACCACGTC
CTAGGCCCAGACCCAGACCCAGGCCTAGACCTAGACCCCGGCCTAGACCCCGTCCTCGTCCTCGTCCAAG

ACCAAGGCCGAGGCCACGCCCTAGGCCCCGTCCACGGCCTCGACCTCGTCCACGACCCAGACCCCGACCT
CGCCCAAGGCCAAGACCACGCCCGCGACCTCGACCGCGGCCACGCCCACGCCCTCGGCCCAGACCACGTC
CTAGGCCCAGACCCAGACCCAGGCCTAGACCTAGACCCCGGCCTAGACCCCGTCCTCGTCCTCGTCCAAG
ACCAAGGCCGAGGCCACGCCCTAGGCCCCGTCCACGGCCTCGACCTCGTCCACGACCCAGACCCCGACCT
CGCCCAAGGCCAAGACCACGCCCGCGA]gtcttccaacgggatccaccggtcgccaccatggtgagcaag
ggcgaggagctgttcaccggggtggtgcccatcctggtcgagctggacggcgacgtaaacggccacaagt
tcagcgtgtccggcgagggcgagggcgatgccacctacggcaagctgaccctgaagttcatctgcaccac
cggcaagctgcccgtgccctggcccaccctcgtgaccaccctgacctacggcgtgcagtgcttcagccgc
taccccgaccacatgaagcagcacgacttcttcaagtccgccatgcccgaaggctacgtccaggagcgca
ccatcttcttcaaggacgacggcaactacaagacccgcgccgaggtgaagttcgagggcgacaccctggt
gaaccgcatcgagctgaagggcatcgacttcaaggaggacggcaacatcctggggcacaagctggagtac
aactacaacagccacaacgtctatatcatggccgacaagcagaagaacggcatcaaggtgaacttcaaga
tccgccacaacatcgaggacggcagcgtgcagctcgccgaccactaccagcagaacacccccatcggcga
cggccccgtgctgctgcccgacaaccactacctgagcacccagtccgccctgagcaaagaccccaacgag
aagcgcgatcacatggtcctgctggagttcgtgaccgccgccgggatcactctcggcatggacgagctgt
acaagtaaagcggccgcgactctaga

GR

gaattcggatgtagaccATG[GGCAGAGGACGCGGTCGGGGACGAGGAAGAGGACGGGGTAGAGGGCGAG
GTCGCGGCCGTGGTAGAGGCAGAGGTCGTGGGAGAGGCAGGGGTCGCGGACGTGGACGGGGAAGGGGACG
AGGTAGAGGCAGGGGACGCGGACGAGGGAGAGGACGGGGCCGTGGTCGAGGGAGAGGTAGAGGCCGAGGT
CGAGGCCGAGGACGAGGACGCGGCAGAGGACGCGGTCGGGGACGAGGAAGAGGACGGGGTAGAGGGCGAG
GTCGCGGCCGTGGTAGAGGCAGAGGTCGTGGGAGAGGCAGGGGTCGCGGACGTGGACGGGGAAGGGGACG
AGGTAGAGGCAGGGGACGCGGACGAGGGAGAGGACGGGGCCGTGGTCGAGGGAGAGGTAGAGGCCGAGGT
CGAGGCCGAGGACGAGGACGCGGCA]gagtcttccaacgggatccaccggtcgccaccatggtgagcaag
ggcgaggagctgttcaccggggtggtgcccatcctggtcgagctggacggcgacgtaaacggccacaagt
tcagcgtgtccggcgagggcgagggcgatgccacctacggcaagctgaccctgaagttcatctgcaccac
cggcaagctgcccgtgccctggcccaccctcgtgaccaccctgacctacggcgtgcagtgcttcagccgc
taccccgaccacatgaagcagcacgacttcttcaagtccgccatgcccgaaggctacgtccaggagcgca
ccatcttcttcaaggacgacggcaactacaagacccgcgccgaggtgaagttcgagggcgacaccctggt
gaaccgcatcgagctgaagggcatcgacttcaaggaggacggcaacatcctggggcacaagctggagtac
aactacaacagccacaacgtctatatcatggccgacaagcagaagaacggcatcaaggtgaacttcaaga
tccgccacaacatcgaggacggcagcgtgcagctcgccgaccactaccagcagaacacccccatcggcga
cggccccgtgctgctgcccgacaaccactacctgagcacccagtccgccctgagcaaagaccccaacgag
aagcgcgatcacatggtcctgctggagttcgtgaccgccgccgggatcactctcggcatggacgagctgt
acaagtaaagcggccgcgactctaga

Online Resource 2.
DPR Sequences. The regions within the [ ] show the DPR core sequences. These sequences are repeated up to full length (see Bennion Callister et al., 2016). For practicality only the core sequence is shown. Green highlighted region shows eGFP tag.

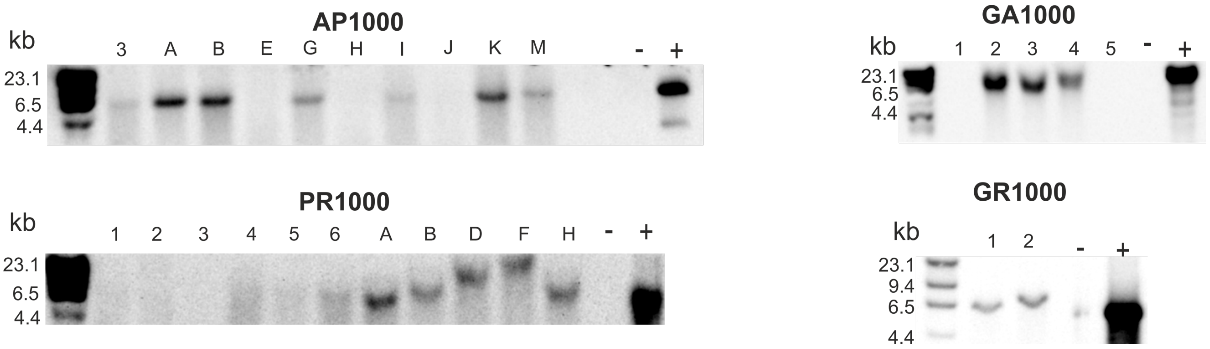

Online Resource 3.

Representative Southern Blots of all potential transformants at ~ 3 months post microinjection. Lanes are labelled with arbitrary labels used during transformant identification. - = negative control (wild type DNA), + = positive control (wild type DNA spiked with positive plasmid).

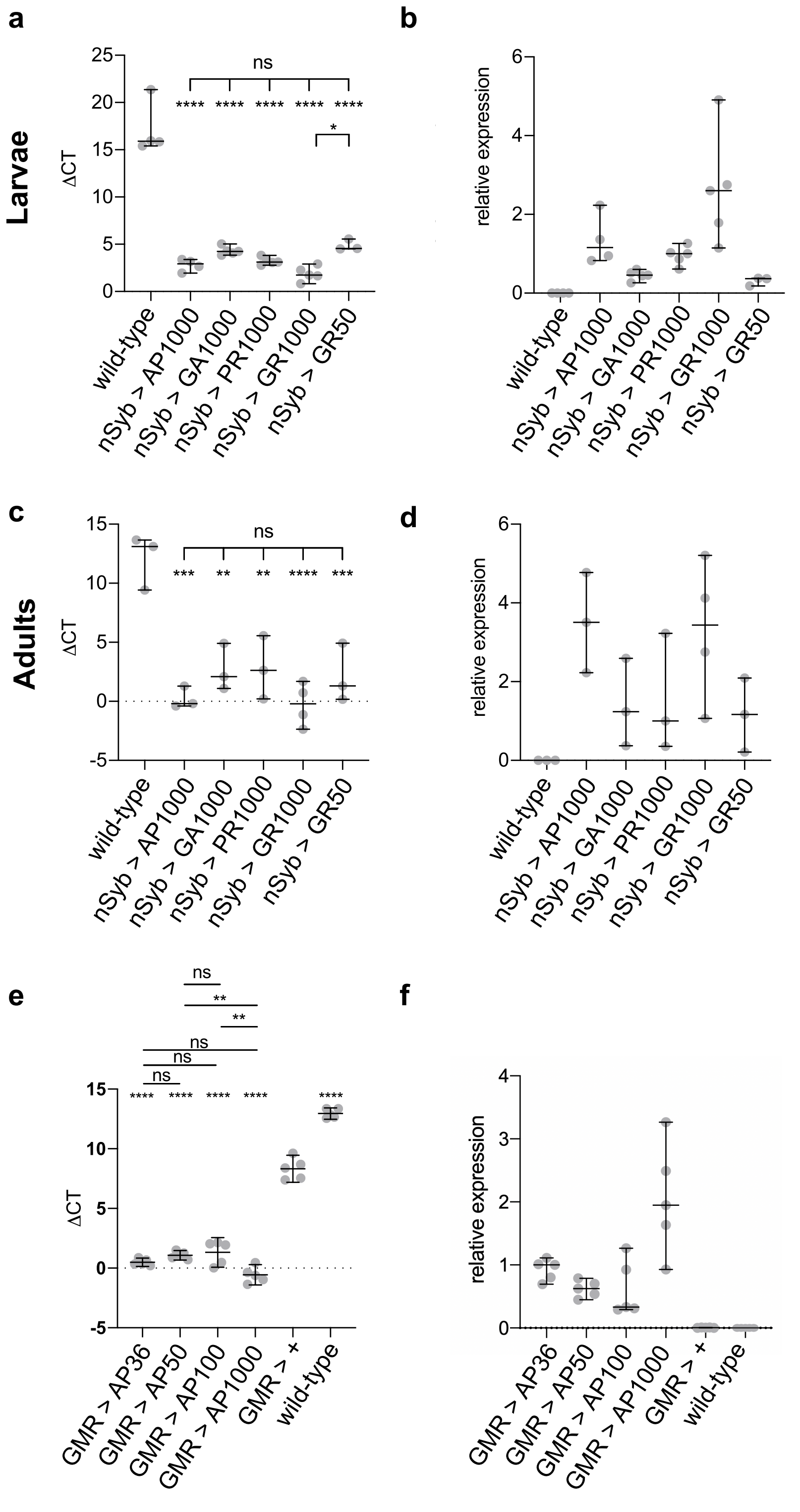

Online Resource 4. Comparison of transcript expression levels of DPR constructs in larval brains and adult heads using realtime qRT-PCR.

Expression of DPR UAS-constructs was detected using primers covering a 5’ region common to all UAS transcript. ΔCTs (a, c, e) and relative transcript expressions (b, d, f) are shown for larval brains (a, b) and adult heads (c-f).

a-d. While expression levels of all pan-neuronally expressed (nSyb-Gal4) 1000 repeat DPRs are significantly different from wild-type controls (no expression) there is no statistical difference in expression levels between different 1000 repeat DPR species in either larval brains (a-b) or adult heads (c-d). Pan-neuronal expression of GR50, used as an established positive control, revealed GR50 had significantly lower expression than GR1000 in larval brains (a) but not adult brains (b).
e-f. Comparison of expression levels between different length models, using AP as a representative model, reveals significantly increased levels of AP1000 compared to AP100 and AP50. All DPR models showed significant greater expression than wild-type and driver only (GMR>+) controls. Constructs were expressed in the fly eye under the control of GMR-Gal4, as pan-neuronal expression of shorter repeats was lethal. Graphs show medians, 95% confidence intervals as whiskers and the datapoints for biological repeats (each derived as mean from technical duplicates).Relative expressions in **b** and **d** are normalised to the median of AP1000, relative expression in **f** is normalised to AP36. Statistical analysis was carried out on ΔCT using ANOVA with posthoc Tukey's multiple comparison. N≥3; * $p<0.05$; ** $p<0.01$, *** $p<0.001$; **** $p<0.0001$; ns: not significant.

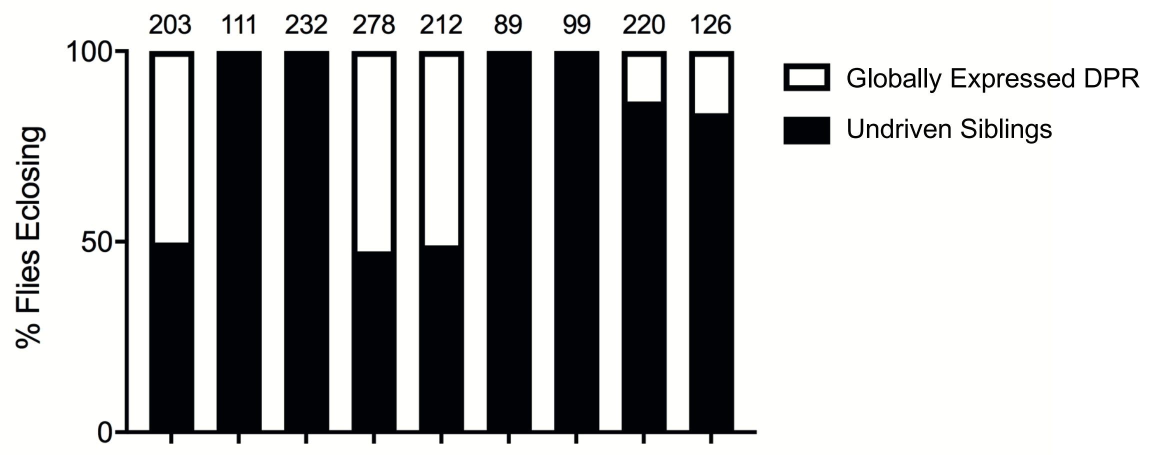

Online Resource 5. Viability Assays for global DPR expression. Global expression (Tubulin-Gal4) of each DPR line results in a lethal phenotype in AP1000 and PR1000 expressing lines and semi-lethality in GR1000 expressing lines

Online Resource 6. Survival Log-Rank (Mantel-Cox) with Bonferroni Correction

	P	P<.05	P<.01	P<.001
**Bonferroni Correction Threshold (K=5):**		**(.01)**	**(.002)**	**(.0002)**
**Wild-type vs GFP**	.3040	N	N	N
**Wild-type vs AP1000**	< .0001	Y	Y	Y
**Wild-type vs GA1000**	.001	Y	Y	N
**Wild-type vs PR1000**	.0735	N	N	N
**Wild-type vs GR1000**	< .0001	Y	Y	Y

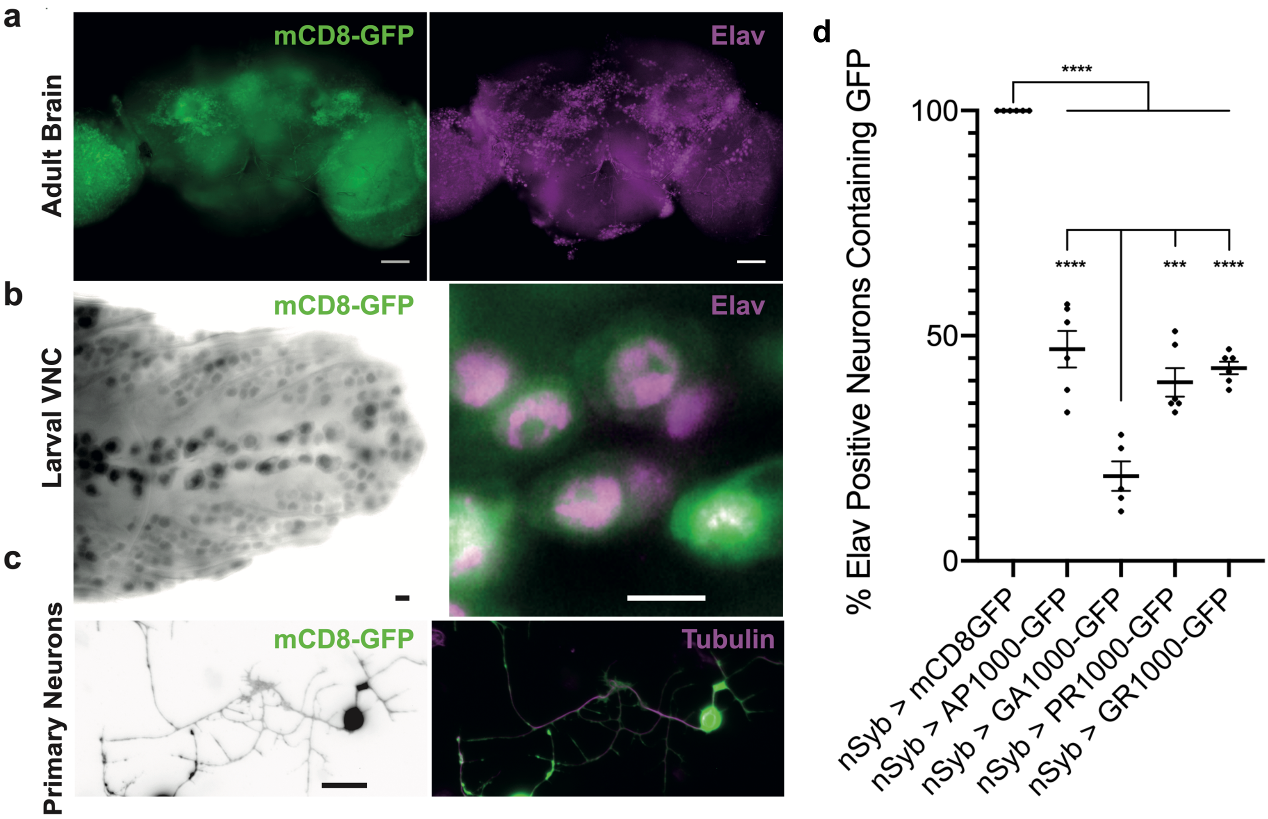

Online Resource 7. Pan-neuronal expression (nSyb-Gal4) of mCD8-GFP in **a** the adult *Drosophila* brain, **b** the central nervous system of third instar larvae and **c** *Drosophila* primary neurons. **d.** Quantification of the number of elav positive neurons within the *Drosophila* adult brain (7 Days Post eclosion) that colocalise with GFP when mCD8-GFP or DPR-GFPs are pan-neuronally expressed (nSyb-Gal4). 300 neurons per brain. **** p <.0001, *** p < .001.

Online Resource 8. Number of flies assayed at each time point for longitudinal negative geotaxis assays 8a

	Days Post Eclosion						
	1	3	7	14	21	28	42
Wild-type	18	17	24	11	16	17	7
mCD8-GFP	-	10	22	14	12	10	10
AP1000	17	17	15	14	19	23	17
GA1000	21	21	15	16	21	18	27
PR1000	16	12	18	14	17	24	20
GR1000	14	10	15	13	10	14	8

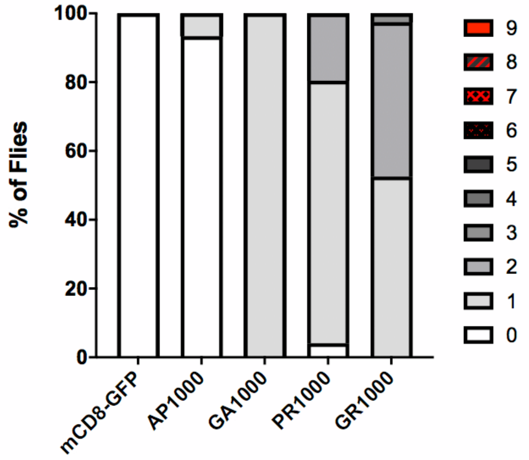

Online Resource 9. Quantification of the eye phenotype in flies expressing DPRs in the *Drosophila* eye (GMR-Gal4) at 29°C
